# Efficacy and safety of traditional Chinese medicine kidney-tonifying and blood-activating therapy in the treatment of vascular dementia: a systematic review and meta-analysis

**DOI:** 10.3389/fneur.2026.1901245

**Published:** 2026-10-20

**Authors:** Mengdie Zhou, Mengqi Che, Chao Zhang, Xiaoyu Yang, Xile Liu, Zhihao Liu, Ju Huang, Hua He

**Affiliations:** 1 Henan University of Traditional Chinese Medicine, Zhengzhou, Henan, China; 2 Chengdu University of Traditional Chinese Medicine, Chengdu, Sichuan, China; 3 Hospital of Chengdu University of Traditional Chinese Medicine, Chengdu, Sichuan, China; 4 Henan Provincial Hospital of Traditional Chinese Medicine (The Second Affiliated Hospital of Henan University of Traditional Chinese Medicine), Zhengzhou, Henan, China

**Keywords:** kidney-tonifying and blood-activating therapy, meta-analysis, systematic review, traditional Chinese medicine, vascular dementia

## Abstract

**Objective:**

This meta-analysis evaluated the efficacy and safety of Kidney-tonifying and Blood-activating Therapy (KTBAT) for vascular dementia (VaD).

**Methods:**

Eight electronic databases were systematically searched from inception to January 12, 2026, for randomized controlled trials (RCTs). Primary endpoints included clinical effectiveness and cognitive scores (MMSE, HDS, CDR). Secondary outcomes focused on neurotrophic factors (BDNF, NGF, GDNF). Study quality was assessed via RoB 1, with data synthesis performed using RevMan 5.4.

**Results:**

Thirty-eight RCTs involving 3,357 participants were identified. KTBAT, whether administered as monotherapy or combined with Western medicine (WMT), significantly increased the clinical effectiveness rate compared to WMT (RR = 1.25, 95% CI: [1.20, 1.31]). Regarding cognition, KTBAT significantly improved MMSE (MD = 3.71, 95% CI: [2.96, 4.47]) and HDS scores (MD = 4.07, 95% CI: [2.63, 5.52]). CDR scores were also lower after intervention (SMD = −0.87, 95% CI: [−1.26, −0.47]). Additionally, KTBAT was associated with higher serum levels of BDNF (SMD = 1.02, 95% CI: [0.13, 1.90]) and NGF (SMD = 0.69, 95% CI: [0.27, 1.12]). GDNF was assessed in only one trial, and its level was also higher in the intervention group. Adverse events (AEs) rates did not differ significantly between KTBAT and WMT (RR = 0.71, 95% CI: [0.32, 1.57], *p* = 0.40), with no serious AEs or deaths reported.

**Conclusion:**

KTBAT appears to be a potentially effective intervention for improving clinical effectiveness and cognitive function in VaD; however, its safety profile remains uncertain. While these findings support KTBAT as a promising candidate for VaD management, further high-quality, multicenter trials are warranted to strengthen the evidence base.

**Systematic review registration:**

https://www.crd.york.ac.uk/prospero/search, identifier CRD420251272344.

## 1 Introduction

As the global population continues to age, dementia has become a major public health concern worldwide. Vascular dementia (VaD), which is caused by cerebrovascular disease, is the second most common type of dementia after Alzheimer’s disease (AD) (1). Previous studies have shown that pure VaD represents about 15% of all dementia cases, affecting an estimated 8.5 million people globally (2). In China, the prevalence of dementia in adults aged 60 years and older is approximately 6.0%, among whom VaD accounts for 1.6% (3). Clinically, VaD is characterized by marked deficits in several cognitive domains, including memory, attention, executive function, and language, often together with focal neurological signs (4). Despite its substantial disease burden, currently, no therapeutic intervention has been definitively established to halt or reverse disease progression (5, 6). Clinical management primarily focuses on slowing cognitive decline, with cholinesterase inhibitors and memantine being the most commonly employed pharmacological agents (7, 8). In addition, international consensus recommends comprehensive control of vascular risk factors, including hypertension and diabetes mellitus, as an important approach to slowing disease progression (9). However, existing pharmacotherapies provide symptomatic effects with limited ability to modify the underlying disease progression. Moreover, their long-term use is often restricted by adverse events (AEs), such as gastrointestinal disturbances and dizziness (10, 11). Due to the limited availability of highly effective clinical options, there is a clear need to investigate complementary and alternative therapeutic strategies, among which traditional Chinese medicine (TCM) represents a promising avenue.

Within TCM theory, VaD is referred to as “dementia (Chi Dai)” or “forgetfulness (Jian Wang)” (12). In terms of etiology and pathogenesis, VaD is linked to “kidney deficiency” (Shen Xu) and “blood stasis” (Xue Yu). Notably, pathological conditions such as metabolic syndrome and coagulation abnormalities (13) align with TCM concepts such as “phlegm-dampness (Tan Shi)” and blood stasis. Both phlegm-dampness and blood stasis have been recognized as established risk factors for cerebrovascular disease and cognitive impairment (14, 15). Blood stasis, meanwhile, describes a state of impaired microcirculation and reduced tissue oxygenation. Therefore, the treatment protocol for VaD in TCM includes the Kidney-tonifying and Blood-activating Therapy (KTBAT) (16). In this review, KTBAT was operationally defined as Chinese herbal interventions—including decoctions, granules, capsules, tablets, and patent medicines—whose primary therapeutic principle is kidney-tonifying and blood-activating, and which contain both kidney-tonifying and blood-activating components for the treatment of VaD. Therefore, pooling these interventions reflects the overall effect of the KTBAT principle, rather than the effect of one specific formula. This therapeutic approach incorporates Chinese herbal medicines (CHMs) that simultaneously tonify the kidneys and activate blood circulation. Classic and modern formulations, such as Buyang Huanwu Tang, Dihuang Yinzi, and Bushen Huoxue Tang, have been widely adopted (17–19). Contemporary pharmacological research indicates that KTBAT exerts multiple effects, including improving cerebral blood flow, having anti-inflammatory properties, antioxidant activity, inhibiting neuronal apoptosis, activating mitophagy, and providing neuroprotection (20–23). KTBAT may also enhance neurotransmitters and offer neuroprotective actions (24, 25). An increasing number of clinical trials have evaluated different KTBAT formulations for VaD (26). However, the methodological quality of these studies varies substantially, and their findings are not fully consistent. Specifically, the effectiveness of TCM for patients with severe dementia remains uncertain, and real-world data are limited.

Given this gap in the literature, TCM has attracted growing attention for its potential use in the management of VaD, and an increasing number of clinical studies have been conducted in this area. Nevertheless, a systematic synthesis and quantitative assessment of the available evidence are still lacking. Therefore, we performed this systematic review and meta-analysis to integrate the existing clinical evidence and evaluate the efficacy and safety of KTBAT for VaD. This study systematically evaluated clinical outcomes and safety of KTBAT in VaD by integrating previous RCTs. This review was conducted in accordance with the PRISMA 2020 guidelines.

## 2 Materials and methods

### 2.1 Protocol registration

This systematic review and meta-analysis was planned, undertaken, and reported in line with the 2020 Preferred Reporting Items for Systematic Reviews and Meta-Analyses (PRISMA) statement. The study protocol was prospectively registered in the International Prospective Register of Systematic Reviews (PROSPERO registration ID: CRD420251272344).

### 2.2 Search strategy

The literature search covered the period from database inception through January 12, 2026. The databases searched were PubMed, Web of Science Core Collection (Web of Science), the Cochrane Central Register of Controlled Trials (CENTRAL), Excerpta Medica Database (Embase), Chinese Biomedical Literature Service System (Sino-Med), China National Knowledge Infrastructure (CNKI), VIP Chinese Journal Database (VIP), and Wanfang Data Knowledge Service Platform (Wanfang).

A combination of Medical Subject Headings (MeSH) and free-text keywords was used to identify records related to the condition, intervention, and study design. Search terms included, but were not limited to, “Dementia, Vascular, Vascular Dementia, Arteriosclerotic Dementia, kidney-tonifying and blood-activating, Bushen Huoxue, and Bushen Huoxue traditional Chinese medicine”. In addition, the reference lists of relevant reviews and eligible studies were manually screened to identify potentially missed articles. No restrictions were applied with regard to publication language or country of origin. The detailed search strategy is presented in Supplementary note 1.

### 2.3 Eligibility criteria

Studies were screened and selected according to the PICOS framework.

Participants (P): Adult patients (aged ≥ 40 years) clinically diagnosed with VaD, regardless of gender, ethnicity, or nationality.

Interventions (I): TCM formulas (including decoctions, granules, or patent medicines) with kidney-tonifying and blood-activating effects, administered either as monotherapy or in combination with Western medicine treatment (WMT).

Comparison (C): Placebo control, WMT (including but not limited to nimodipine, donepezil, huperzine A, etc.), or no treatment (blank control).

Outcomes (O):

Primary outcome: The clinical effectiveness rate at the end of treatment is determined based on criteria such as MMSE score changes or global symptom assessment.

Secondary outcomes: Mini-Mental State Examination (MMSE); Hasegawa Dementia Scale (HDS); Clinical Dementia Rating Scale (CDR); brain-derived neurotrophic factor (BDNF); nerve growth factor (NGF); glial cell line-derived neurotrophic factor (GDNF); AEs.

Study design (S): Randomized controlled trials (RCTs).

### 2.4 Exclusion criteria

Studies were excluded if they met any of the following criteria: (1) duplicate reports or overlapping datasets; (2) review articles, including systematic reviews and meta-analyses, as well as basic research or animal studies; (3) studies where the experimental or control group received additional interventions (e.g., acupuncture, rehabilitation therapy, physical therapy) beyond the predefined treatment protocols; (4) studies with incomplete data or unreported key outcome indicators; (5) the full text could not be retrieved or no extractable data were reported; (6) uncontrolled studies lacking a parallel control group.

### 2.5 Selection process

Two reviewers (Zhou and Che) independently performed study selection in two steps. Titles and abstracts were first screened, followed by full-text assessment against the eligibility criteria. Any disagreement was resolved by discussion, and a third reviewer was consulted when consensus could not be reached. Duplicate records were identified and removed using EndNote 2025 (Clarivate Analytics, USA), with both automated and manual deduplication procedures applied. The entire selection workflow, from identification to final inclusion, is depicted in a PRISMA 2020 flow diagram.

### 2.6 Data collection process

Data extraction was carried out independently by the same two reviewers using a pretested standardized form. The following information was collected from each eligible study: first author, publication year, country, sample size, participant characteristics, intervention and comparator details, treatment duration, outcome definitions, numerical results, and AEs. When relevant information was missing or unclear, attempts were made to contact the corresponding authors for clarification. All data were entered into Microsoft Excel for organization and analysis. The extracted information was then cross-checked, and any discrepancies were resolved through discussion or, if necessary, by consultation with a third reviewer.

### 2.7 Study risk of bias assessment

The methodological quality of the included RCTs was independently evaluated by two reviewers (Zhou and Che) using the Cochrane Risk of Bias tool version 1 (RoB 1). The assessed domains included random sequence generation, allocation concealment, blinding of participants and personnel, blinding of outcome assessment, incomplete outcome data, selective reporting, and other bias. Each domain was categorized as low, unclear, or high risk of bias, and an overall judgment was assigned to each study accordingly. Discrepancies were settled through discussion or, when needed, by adjudication from a third reviewer.

### 2.8 Data synthesis and statistical analysis

Statistical analyses were performed using Review Manager software (RevMan version 5.4; The Nordic Cochrane Centre). Meta-analysis was conducted when at least two studies reported sufficiently comparable interventions, outcomes, and study designs. Statistical heterogeneity was examined using Cochran’s Q test and quantified with the *I*^2^ statistic.

For dichotomous outcomes, such as the clinical effectiveness rate, pooled effect sizes were expressed as risk ratios (RRs) with 95% confidence intervals (CIs). The clinical effectiveness rate was extracted as a dichotomous outcome (effective vs. ineffective) according to the definition provided in each original study. For continuous outcomes, mean differences (MDs) with 95% CIs were calculated when the same measurement scale was used across studies; otherwise, standardized mean differences (SMDs) with 95% CIs were applied.

A fixed-effect model was used when heterogeneity was low (*I*^2^ ≤ 50% and *p* ≥ 0.10 for the *Q*-test), indicating that the observed variation across studies was likely due to chance. When heterogeneity was moderate to substantial (*I*^2^ > 50% and/or *p* < 0.10), a random-effects model was adopted to account for expected clinical and methodological differences across studies.

When substantial heterogeneity was identified, potential sources were explored through subgroup analyses and sensitivity analyses. Differences in participant characteristics, intervention dosage forms and treatment duration were considered as possible contributors to heterogeneity. A two-sided *p* value < 0.05 was considered statistically significant for pooled effect estimates.

For crossover trials, only first-period data were extracted. When first-period data were available, they were treated as parallel-group data and included in the primary quantitative synthesis. When only combined-period data or per-period data with repeated measures were reported, the trial was not included in the primary quantitative synthesis and was summarised narratively. The same rule was applied to safety outcomes.

### 2.9 Subgroup analyses

To further explore potential sources of heterogeneity and maintain consistency with the predefined analytical framework, subgroup analyses were planned according to the following factors: treatment duration, categorized based on the intervention courses reported in the included studies; dosage form, such as different pharmaceutical or preparation forms of the intervention. Subgroup findings were interpreted cautiously due to the observational nature of between-subgroup comparisons.

### 2.10 Sensitivity analyses

Sensitivity analyses were undertaken to evaluate the stability of the pooled findings. Specifically, leave-one-out analyses were performed by sequentially excluding one study at a time and recalculating the combined effect size to determine whether individual studies influenced the overall estimates or heterogeneity. Additional sensitivity analyses were planned to examine the impact of study quality, sample size, and model choice.

### 2.11 Assessment of reporting biases

When a meta-analysis included at least 10 studies, funnel plots were generated to evaluate possible small-study effects and publication bias. However, the limited power of these methods was taken into account when the number of studies was small. It should be noted that an asymmetrical funnel plot does not necessarily indicate the presence of publication bias.

## 3 Results

### 3.1 Study selection

A total of 2,827 records were retrieved from eight electronic databases. After 1,173 duplicate entries were removed, 1,654 unique records remained for subsequent screening. Following evaluation of the titles and abstracts, 1,308 records were excluded because they did not satisfy the predefined eligibility criteria. The full texts of the remaining 346 articles were then obtained and assessed for eligibility; among them, three studies were excluded because the full texts were unavailable. Ultimately, 343 publications were independently evaluated by two researchers using RoB 1, resulting in the inclusion of 38 RCTs (27–64). The study selection process is presented in Figure 1.

**Figure 1:**
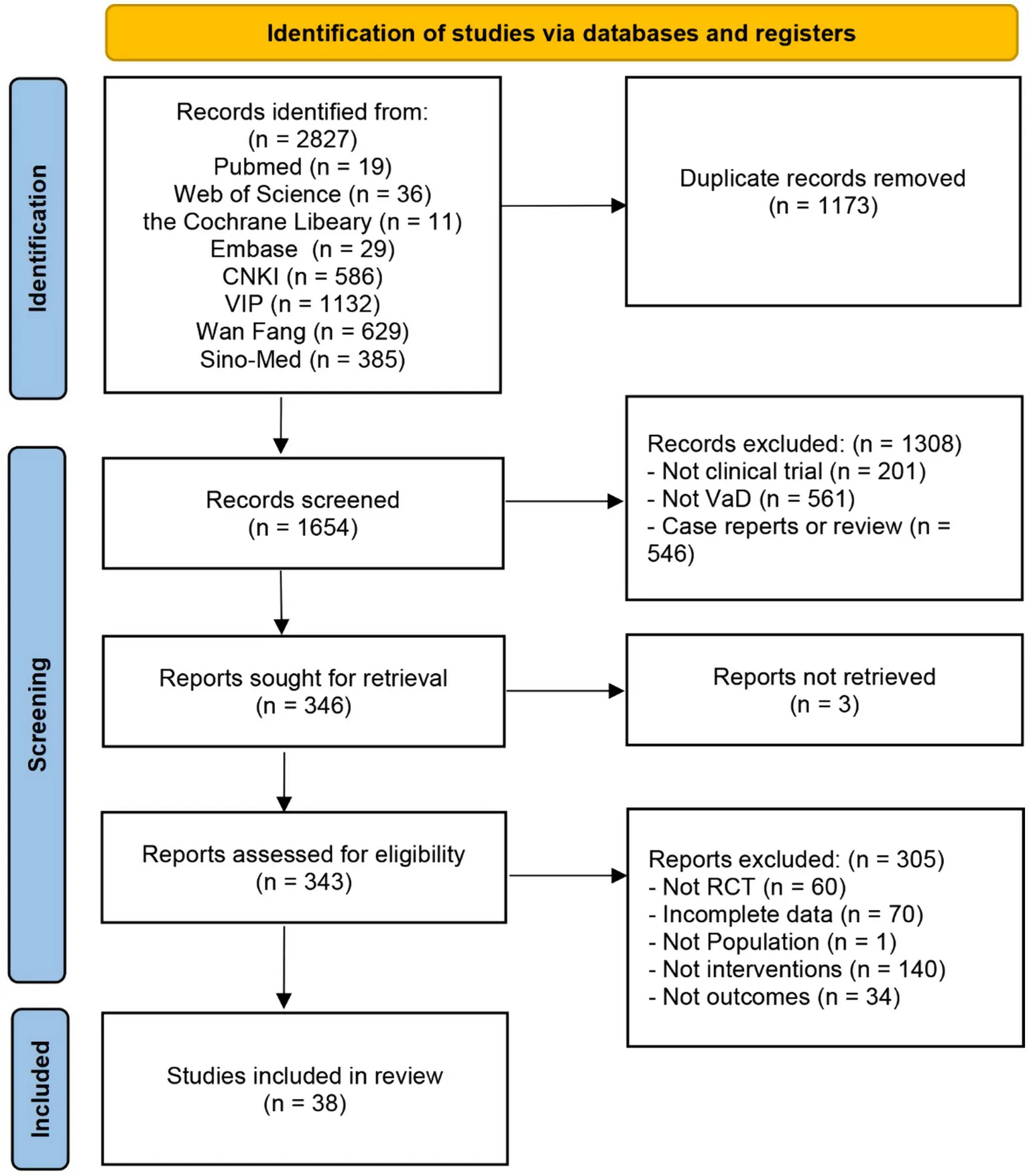
Study selection flowchart. “n” represents the number of articles retrieved.

### 3.2 Study characteristics

A total of 38 articles (27–64) published between 2003 and 2025 were included in this review. All of the studies were conducted in China and reported in Chinese. The overall sample comprised 3,357 participants, with 1,692 assigned to the intervention group and 1,665 to the control group. The treatment period varied from 1 to 6 months. In all included trials, based on conventional Western medicine treatments (CWMT), such as antihypertensive and hypoglycemic therapies, the intervention group received TCM prescriptions or patent medicines focused on KTBAT, which were available in various formulations including capsules, granules, and tablets. Meanwhile, the control group received Western medicine treatment (WMT) or placebos, including donepezil, memantine, oxiracetam, and butylphthalide soft capsules. A clinical effectiveness rate was reported as an outcome measure in all included RCTs.

The included studies covered three comparison patterns. In 22 RCTs, KTBAT was combined with WMT, involving 962 patients in the intervention groups and 943 patients in the control groups. In 13 RCTs, KTBAT was used as monotherapy and compared with WMT, including 667 patients in the intervention groups and 659 patients in the control groups. In the remaining 3 RCTs, KTBAT was compared with placebo. These three crossover trials included 126 participants (63 in each group). Three placebo-controlled trials were crossover trials with non-poolable data. Additionally, nine studies documented the incidence of AEs; however, three crossover trials were not pooled for AEs, leaving six trials in the quantitative AE synthesis. The detailed characteristics of the included RCTs are summarized in Table 1.

**Table 1:** Features of the included studies.

Author	Country	Study period	Patients (N)		Age		Females		Treatments		Hospital stay	Outcomes
			T	C	T	C	T	C	T	C		
Dou and Gao (27)	China	2018–2021	47	47	NR	NR	22	23	Naoxintong Capsule + Piracetam Tablets + CWMT	Piracetam Tablets + CWMT	3 months	①②
Guo et al. (28)	China	2013–2016	60	60	65.12 ± 5.36	64.83 ± 4.97	34	32	Kidney-invigorating Blood-activating Tablets	Citicoline Tablets	8 weeks	①②③⑧
Han et al. (29)	China	2018–2020	54	54	64.82 ± 4.00	64.25 ± 4.13	24	26	Naoxintong Capsule + Nimodipine Tablets + CWMT	Nimodipine Tablets + CWMT	8 weeks	①②④⑥⑦
Hu et al. (30)	China	2019–2022	50	50	67.83 ± 8.03	66.99 ± 9.77	25	24	Dihuang Yinzi	Piracetam Tablets	12 weeks	①②
Hu and Yu (31)	China	2008–2010	37	30	65.06 ± 7.75	67.12 ± 8.51	15	13	Piracetam+FuYuanDecoction	Piracetam	2 months	①
Huang (32)	China	2017–2019	53	50	61.50 ± 5.80	60.90 ± 7.10	24	20	Naoxintong Capsule + Nimodipine Tablets + CWMT	Nimodipine Tablets + CWMT	6 months	①②③⑥⑧
Jiang and Xu (33)	China	2011–2012	34	32	60.38 ± 2.54	60.56 ± 2.34	13	13	Aricept + Naoxintong Capsule + CWMT	Aricept + CWMT	8 weeks	①②⑤
Jiang (34)	China	2003–2006	30	30	65.50 ± 4.20	64.70 ± 5.00	12	13	Naoxintong Capsule + Duxil	Duxil	2 months	①③
Jin et al. (35)	China	NR	36	36	73.94 ± 7.77	73.94 ± 7.77	18	18	Jiannao Yizhi Capsule	Placebo Capsules	12 weeks	①②③⑧
Li (36)	China	2017–2020	48	48	70.05 ± 3.87	68.82 ± 3.61	15	17	CWMT + Bushen Huoxue Yizhi Decoction	Butylphthalide + CWMT	6 months	①②④
Li et al. (37)	China	2012–2014	57	53	70.40 ± 5.70	69.50 ± 6.20	22	21	Donepezi + CWMT + Dihuang Yinzi Capsule	Donepezi + CWMT	180 days	①②
Li (38)	China	2014–2016	119	119	67.32 ± 7.43	66.85 ± 3.96	51	47	Naoxintong Capsule + CWMT	Nimodipine Tablets + CWMT	6 months	①②③⑤⑧
Liu (39)	China	2016–2018	88	88	73.90 ± 6.50	74.40 ± 6.60	41	39	Bushen Yizhi Decoction	Butylphthalide Capsules	6 months	①②④
Lu et al. (40)	China	2018–2020	48	48	65.71 ± 11.46	64.32 ± 10.76	23	22	Naoxintong Capsule + Donepezil Hydrochloride + CWMT	Donepezil Hydrochloride + CWMT	2 months	①②⑥
Ma et al. (41)	China	2015–2017	46	46	66.73 ± 5.10	66.58 ± 4.95	20	21	Bushen Huoxue Decoction + CWMT + Rehabilitation Nursing Intervention	Donepezil + CWMT + Rehabilitation Nursing Intervention	90 days	①②③
Man et al. (42)	China	2016–2018	47	47	60.48 ± 6.73	60.61 ± 6.85	NR	NR	Naoxintong Capsule + Memantine Hydrochloride Tablet	Memantine Hydrochloride Tablet	2 months	①②
Peng and Deng (43)	China	NR	28	28	68.50 ± 7.85	64.30 ± 8.14	10	9	Huayu Yizhi mixture	Hydergine	2 months	①②③
Shao (44)	China	NR	31	24	NR	NR	10	9	Xingnao Fusu Decoction	Hydergine	2 months	①②
Sheng et al. (45)	China	2005–2008	45	45	66.25 ± 7.16	68.13 ± 8.16	17	19	Naoxintong Capsule	Duxil	12 weeks	①②③
Tang et al. (46)	China	2001–2004	40	40	65.30 ± 7.80	64.60 ± 5.60	17	15	Bushen Huoxue Decoction	Nimodipine + Duxil	3 months	①②⑤
Tao et al. (47)	China	2013–2015	46	46	70.20 ± 5.30	69.80 ± 5.70	17	19	Yizhi Decoction + Oxiracetam Injection	Oxiracetam Injection	3 weeks	①②⑤
Wnag et al. (48)	China	NR	9	9	73.94 ± 7.77	73.94 ± 7.77	2	2	Shenlong Decoction	Placebo	3 months	①②③⑤⑧
Wang et al. (49)	China	2000–2004	18	18	73.14 ± 6.71	73.14 ± 6.71	6	6	Shen Long Decoction	Placebo	3 months	①⑧
Wang et al. (50)	China	2019–2022	31	31	60.94 ± 6.38	59.75 ± 5.72	12	13	Bushen Huazhuo Huoxue Decoction + Memantine Hydrochloride	Memantine Hydrochloride	24 weeks	①②⑥⑧
Wang (51)	China	2010–2013	30	30	NR	NR	NR	NR	Xinjia Dihuang Yinzi + Aspirin enteric-coated tablets + Piracetam Tablets	Aspirin enteric-coated tablets +Piracetam Tablets	3 months	①②
Wan g et al. (52)	China	2013–2015	38	38	71.20 ± 5.40	70.40 ± 6.20	16	13	Tongqiao Huoxue Decoction + Oxiracetam Capsules	Oxiracetam Capsules	1 month	①②
Wu et al. (53)	China	2023–2024	32	32	NR	NR	NR	NR	Dihuang Yinzi + Donepezil Hydrochloride Tablets + CWMT	Donepezil Hydrochloride Tablets + CWMT	90 days	①②
Wu et al. (54)	China	2017–2019	50	50	67.05 ± 5.92	66.73 ± 5.89	22	21	Kaixin powder and Tongqiao Huoxue tang	Donepezil tablets + Nimodipine tablets	16 weeks	①②
Yang et al. (55)	China	2022–2023	38	37	67.13 ± 8.17	67.11 ± 8.16	15	17	CWMT + Jiawei Dihuang Yinzi granules	CWMT	3 months	①②
Yang et al. (56)	China	2016–2017	42	40	66.30 ± 2.50	65.70 ± 2.60	22	19	Naoxintong Capsule + Nimodipine Tablets + CWMT	Nimodipine Tablets + CWMT	3 months	①②
Yao et al. (57)	China	2014–2016	51	51	64.01 ± 7.75	63.51 ± 7.89	21	22	Donepezi + CWMT + Gunao Yizhi Tang	Donepezi + CWMT	3 months	①②
Yao and Yang (58)	China	2017–2019	39	39	65.34 ± 3.56	66.18 ± 3.47	16	17	Naoxintong Capsule + Nimodipine Tablets + CWMT	Nimodipine Tablets + CWMT	6 months	①③
Yu (59)	China	2016–2017	44	44	68.50 ± 5.20	67.30 ± 5.40	18	17	Wenshen Huoxue Recipe + Oxiracetam Capsules + CWMT	Oxiracetam Capsules + CWMT	3 months	①
Yuang and Zhang (60)	China	2021–2024	50	50	69.02 ± 4.32	68.44 ± 4.16	25	24	CWMT + Bushen Huayu Fang	Nimodipine + CWMT	2 months	①②⑤⑥⑧
Zhao (61)	China	2021–2023	49	49	67.44 ± 6.78	67.52 ± 6.55	23	22	Tongqiao Huoxue Decoction + Butylphthalide Soft Capsules + CWMT	Butylphthalide Soft Capsules + CWMT	30 days	①
Zheng et al. (62)	China	2006–2009	27	26	67.8 ± 5.7(60)	67.8 ± 5.7(60)	24 (60)	24 (60)	Naoxintong Capsule + CWMT	Nimodipine Tablets + CWMT	3 months	①②
Zhong et al. (63)	China	2017–2018	35	35	63.31 ± 7.83	64.14 ± 6.48	14	16	Bushen Huoxue Decoction + CWMT	Donepezil + CWMT	60 days	①②⑧
Zhu (64)	China	2012–2014	65	65	NR	NR	29	31	Naoxintong Capsule + Nimodipine Tablets + CWMT	Nimodipine Tablets + CWMT	6 months	①②

AEs: adverse events; BDNF: brain-derived neurotrophic factor; C: control group; CWMT: conventional Western medicine treatments; CDR: Clinical Dementia Rating Scale; GDNF: glial cell line-derived neurotrophic factor; HDS: Hasegawa Dementia Scale; MMSE: Mini-Mental State Examination; NGF: nerve growth factor; NR: not reported; T: treatment group.

Outcome measures: ① Clinical effectiveness rate; ② MMSE; ③ HDS; ④ CDR; ⑤ BDNF; ⑥ NGF; ⑦ GDNF; ⑧AEs.

Analysis of the CHMs dosage forms utilized in the treatment groups revealed that decoctions were the most prevalent (*n* = 20), followed by capsules (*n* = 15). Other dosage forms, including granules, tablets, and mixtures, were each employed in a single study. Among the 38 RCTs, 25 of the most frequently appearing herbal decoction ingredients included Chuanxiong Rhizoma, Rehmanniae Radix Praeparata, *Polygonum multiflorum*, and Corni Fructus, among others. Although all studies applied the Kidney-tonifying and Blood-activating principle, the specific herbal compositions and dosage regimens varied across trials. The specific compositions and formulations of the KTBAT prescriptions identified in the included literature are summarized in Supplementary Table 2.

### 3.3 Risk of bias in studies

We evaluated the quality of the 38 selected studies using the Cochrane Collaboration Risk of Bias tool (Figure 2).

**Figure 2:**
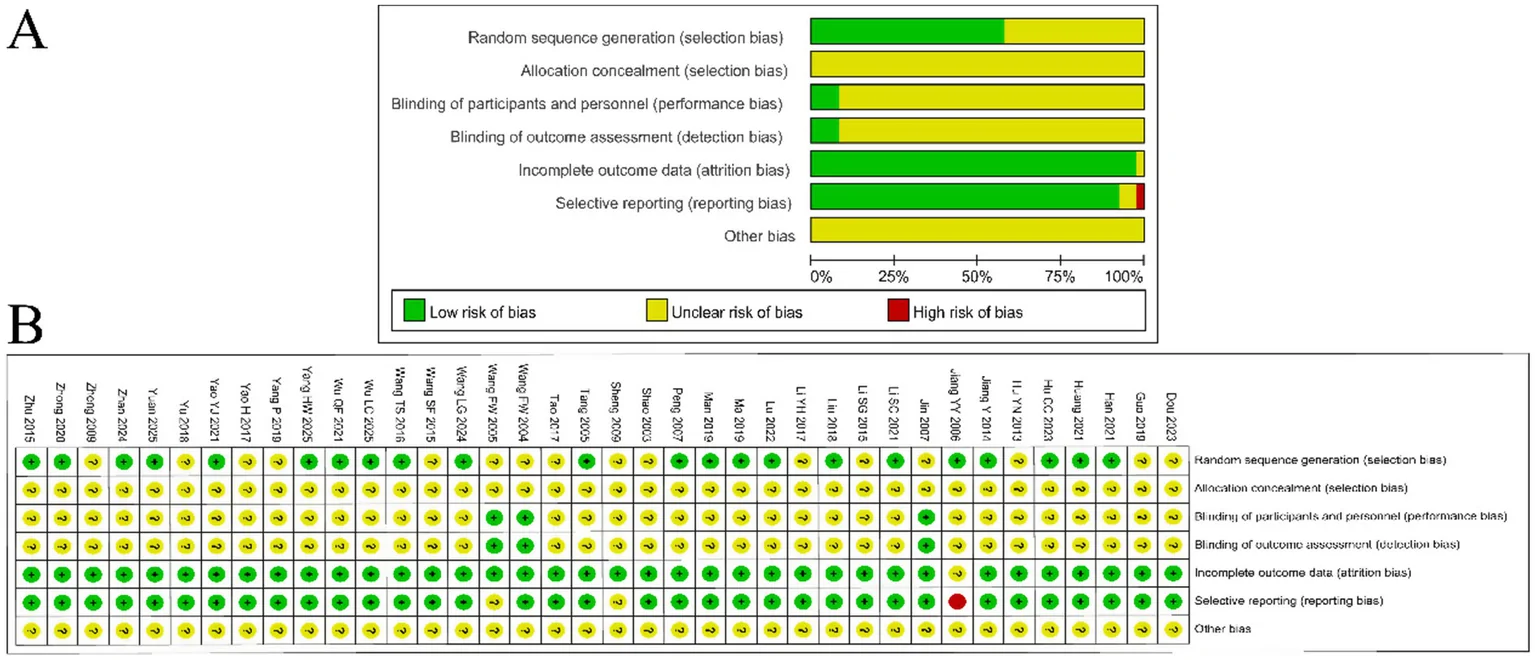
Risk of bias for included RCTs. **(A)** Risk of bias graph; **(B)** Risk of bias summary.

#### 3.3.1 Randomization process

Twenty-two studies used a random number table to generate the allocation sequence and reported comparable baseline characteristics between groups; these studies were therefore considered to have a low risk of bias. For the remaining studies, however, the available information was insufficient to determine whether the randomization process was adequately implemented, and their risk of bias could not be confidently judged.

#### 3.3.2 Allocation concealment

Regarding allocation concealment, none of the 38 included trials provided sufficient details on concealment methods (e.g., sealed opaque envelopes and central allocation). Although all trials were described as randomized, the absence of explicit concealment information precluded a judgment of low risk. Therefore, all 38 trials were judged as having an “unclear risk of bias” for allocation concealment, and no trial was assessed as low risk of bias.

#### 3.3.3 Blind methods

Three articles (35, 48, 49) were judged as having a low risk of bias because they clearly described the blinding process. By comparison, the remaining trials were assigned an unclear risk of bias owing to inadequate reporting of blinding for participants, personnel, and outcome assessors.

#### 3.3.4 Missing outcome data

For incomplete outcome data, one study (34) was rated as high risk because prespecified outcome measures were not fully reported. The other studies either had no missing data or addressed missing data appropriately, and were thus classified as “low risk of bias”.

#### 3.3.5 Selective reporting

In terms of selective reporting, one trial (34) was judged as high risk due to incomplete reporting of prespecified outcome measures. Two studies (45, 49) were regarded as having an unclear risk of bias due to insufficient reporting of outcome measures. The remaining trials showed no selective bias in results, thus assessed as “low risk of bias”.

#### 3.3.6 Other potential sources of bias

No other potential sources of bias were identified across the 38 included trials.

### 3.4 Efficacy outcomes

#### 3.4.1 Clinical effectiveness rate

All 38 included studies (27–64), encompassing a total of 3,357 patients, reported clinical effectiveness rate. Although the criteria for clinical effectiveness differed slightly across studies, they generally required the disappearance or marked alleviation of the main clinical symptoms. It is important to note that this outcome reflects a composite, symptom-based measure of global improvement (Supplementary Table 3).

For 22 RCTs comparing KTBAT combined with WMT versus WMT, a fixed-effect model showed no significant heterogeneity (*I*^2^ = 0%, *p* = 0.68). The pooled results indicated that the combination therapy was associated with a higher clinical effectiveness rate than WMT alone (RR = 1.25, 95% CI: [1.20, 1.31], *p* < 0.00001). Similarly, fixed-effect model analyses were conducted for 13 studies utilizing KTBAT as monotherapy versus WMT. The pooled results indicated low heterogeneity (*I*^2^ = 27%, *p* = 0.17). The pooled analysis showed that KTBAT was associated with a higher clinical effectiveness rate than WMT (RR = 1.26, 95% CI: [1.19, 1.34], *p* < 0.00001). The three placebo-controlled crossover trials (35, 48, 49) were not included in the quantitative synthesis (Figure 3).

**Figure 3:**
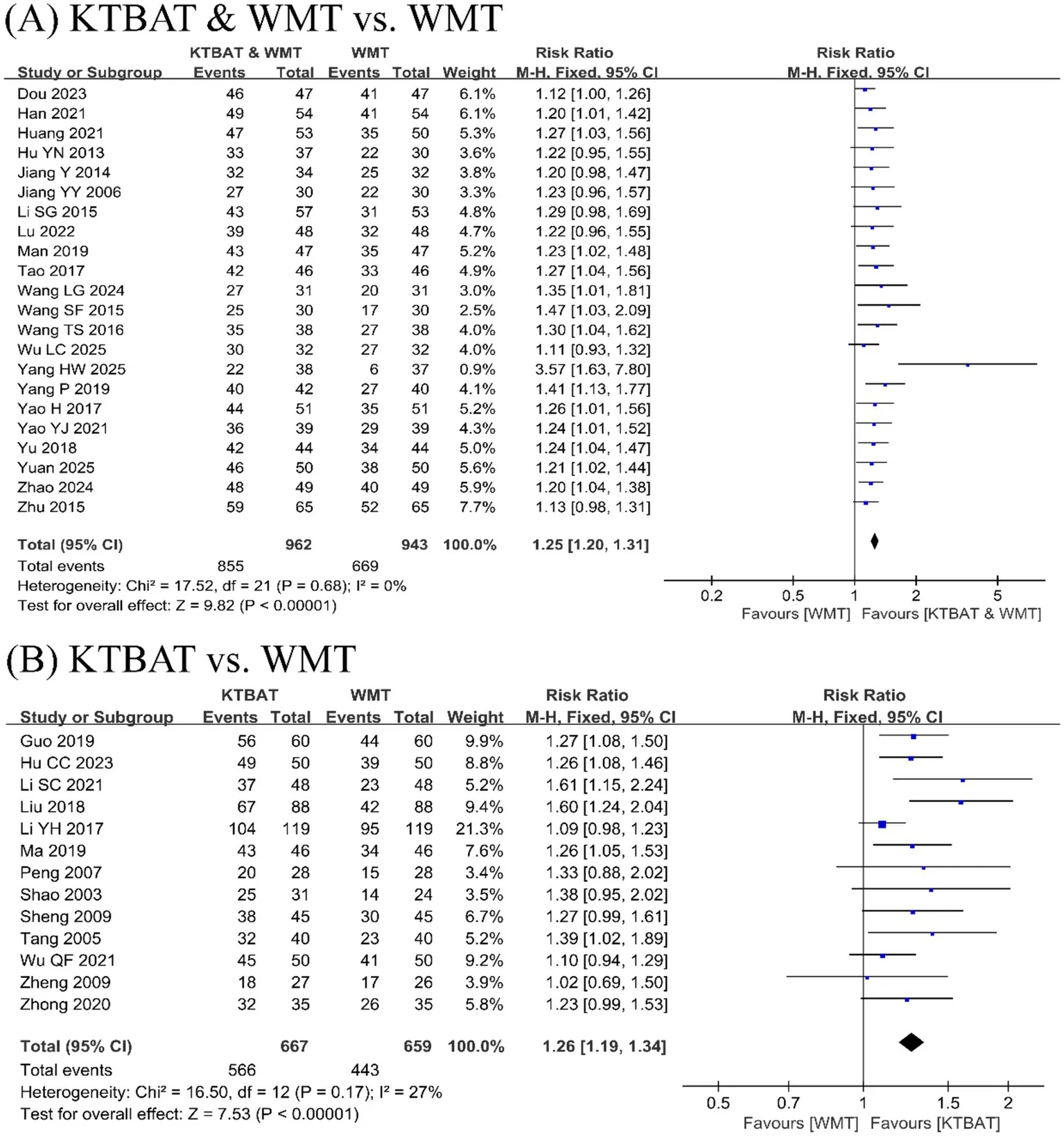
Forest plots of clinical effectiveness rate comparing KTBAT with WMT in VaD treatments. **(A)** KTBAT combined with WMT for a subgroup efficacy analysis compared to using WMT alone. **(B)** A comparative analysis of the efficacy between KTBAT and WMT.

#### 3.4.2 MMSE

A total of 32 RCTs reported MMSE scores as a measure of cognitive improvement (27–30, 32, 33, 35–48, 50–57, 60, 62–64). The pooled analysis revealed substantial heterogeneity among the studies. Consequently, a random-effects model was applied for the analysis.

The pooled estimates for MMSE scores, reflecting cognitive status, were analyzed across three comparison patterns. In the 17 studies evaluating KTBAT combined with WMT, the random-effects meta-analysis showed no significant heterogeneity (*I*^2^ = 0%, *p* < 0.00001). KTBAT combined with WMT achieved a markedly higher improvement in MMSE scores (MD = 3.71, 95% CI: [2.96, 4.47], *p* < 0.00001). Similarly, when KTBAT monotherapy was compared to WMT (13 studies), a significant therapeutic advantage was observed (MD = 3.59, 95% CI: [2.60, 4.58], *I*^2^ = 87%, *p* < 0.00001). The two placebo-controlled crossover trials (35, 48) were not included in the quantitative synthesis (Figure 4).

**Figure 4:**
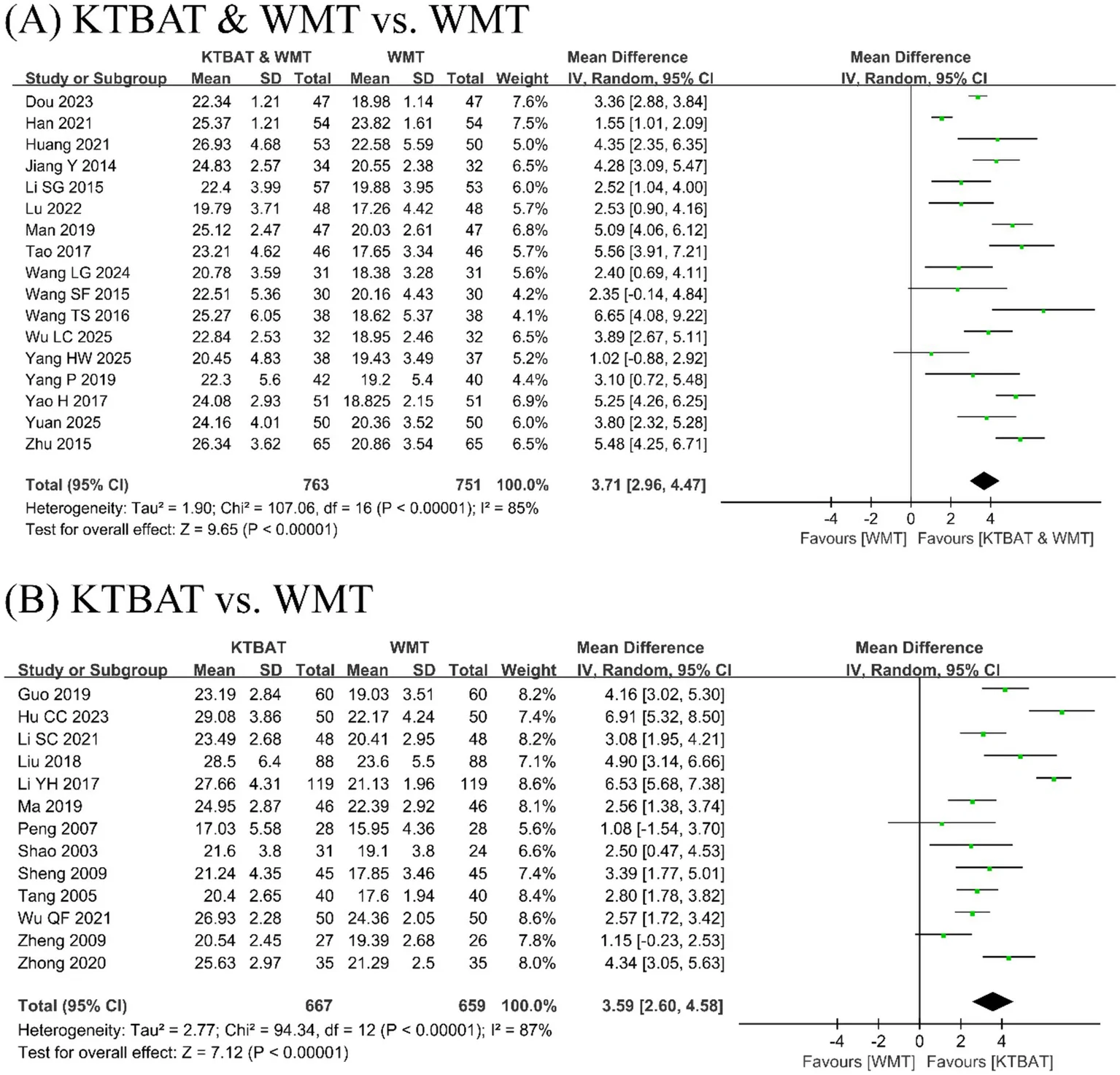
Forest plots of MMSE comparing KTBAT with WMT in VaD treatments. **(A)** KTBAT combined with WMT for a subgroup efficacy analysis compared to using WMT alone. **(B)** A comparative analysis of the efficacy between KTBAT and WMT.

Overall, the method of KTBAT, when used as an adjunctive therapy in combination with WMT, has the highest intensity in enhancing cognitive function and the most extensive evidence base.

To explore the sources of heterogeneity, subgroup analyses were performed according to dosage forms and treatment durations. In the combination therapy group, capsules (MD = 3.92, 95% CI: [3.13, 4.70]) and decoctions (MD = 3.87, 95% CI: [2.44, 5.30]) emerged as more effective forms, whereas granules showed no statistical difference (*p* = 0.29) (Supplementary Figure 1). For treatment duration, therapeutic effects were consistent from 1- to 6-month range, with the most pronounced MD observed at 1 month (MD = 5.88, 95% CI: [4.49, 7.26]), though 6 months interventions provided more robust evidence for long-term efficacy (MD = 3.72, 95% CI: [2.09, 5.34]) (Supplementary Figure 2). In the KTBAT monotherapy group, tablets and capsules were associated with greater improvement than mixtures, and the 6-month treatment subgroup showed the largest MMSE gain (MD = 4.85, 95% CI: [2.52, 7.19]) (Supplementary Figures 3, 4).

#### 3.4.3 HDS

Ten trials (28, 32, 34, 35, 38, 41, 43, 45, 48, 58) reported HDS scores as an outcome measure. After excluding the two crossover trials (35, 48), eight trials encompassing 837 patients were included in the quantitative synthesis. In the adjuvant therapy analysis, three studies evaluating KTBAT combined with WMT showed improved HDS scores (MD = 4.07, 95% CI: [2.63, 5.52], *p* < 0.00001). However, moderate heterogeneity was observed (*I*^2^ = 75%). After excluding (32) in a sensitivity analysis, heterogeneity was no longer detected (*I*^2^ = 0%), and the pooled result remained significant (MD = 4.76, 95% CI: [4.00, 5.52], *p* < 0.00001) (Supplementary Figure 5). In five studies evaluating KTBAT monotherapy, HDS scores also improved significantly (MD = 3.94, 95% CI: [0.83, 7.05], *p* = 0.01). However, substantial heterogeneity was observed (*I*^2^ = 98%), possibly due to clinical variation across studies (Figure 5). Overall, KTBAT combined with WMT provided the most consistent evidence among the three comparison patterns.

**Figure 5:**
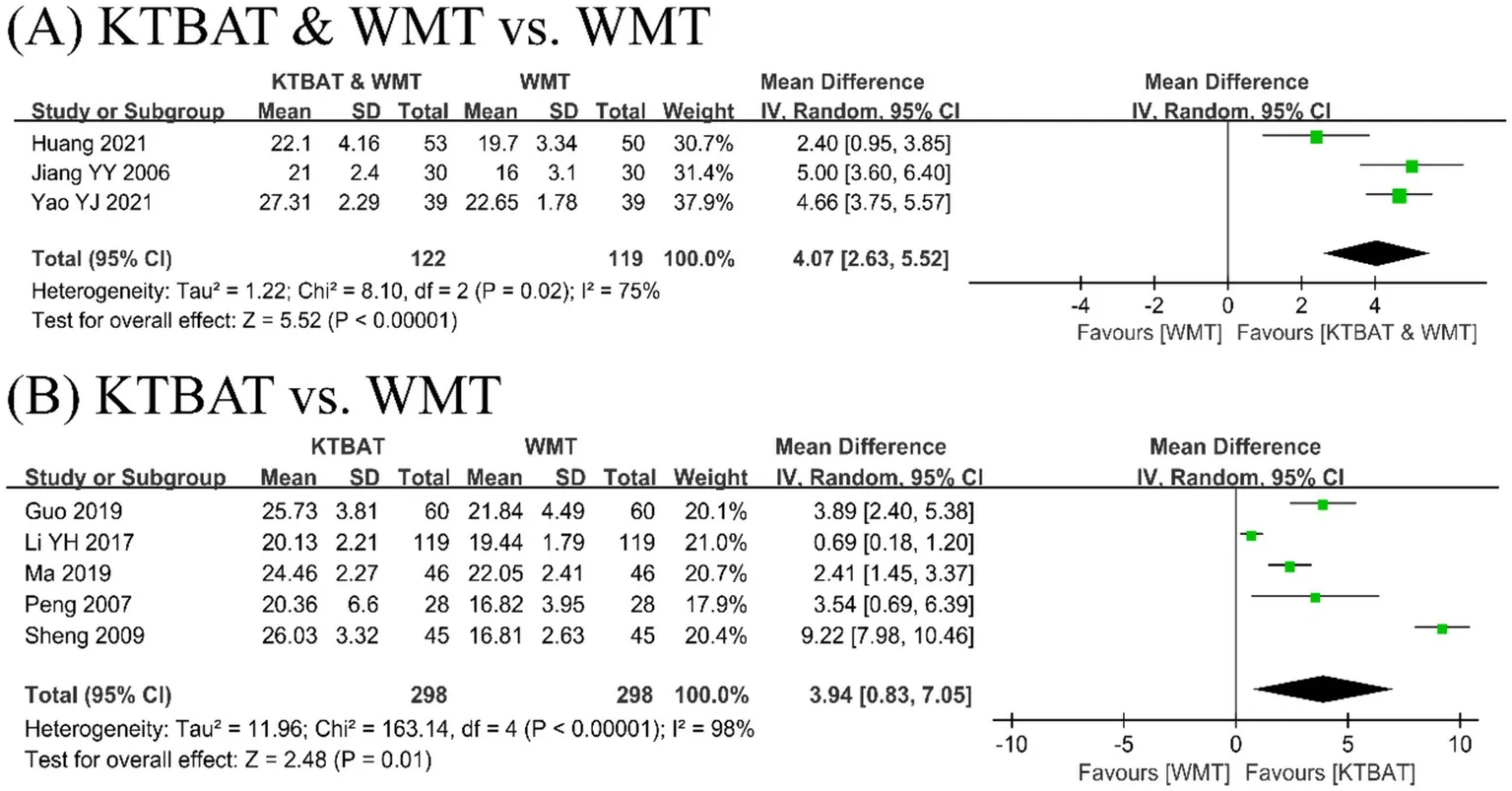
Forest plots of HDS comparing KTBAT with WMT in VaD treatments. **(A)** KTBAT combined with WMT for a subgroup efficacy analysis compared to using WMT alone. **(B)** A comparative analysis of the efficacy between KTBAT and WMT.

Subgroup analyses were conducted in the KTBAT monotherapy group according to dosage form and treatment duration. Tablets and herbal decoctions were associated with significant improvements in HDS scores (*p* < 0.00001), and the mixture subgroup also showed a favorable effect. By contrast, the capsule subgroup (2 studies) showed substantial heterogeneity (*I*^2^ = 99%) and did not reach statistical significance (*p* = 0.25) (Supplementary Figure 6).

For treatment duration, the 2-month subgroup (2 studies) showed a stable and significant benefit (MD = 3.81, 95% CI: [2.49, 5.14]) with no heterogeneity (*I*^2^ = 0%, *p* < 0.00001). The 3-month subgroup (2 studies) showed substantial heterogeneity (*I*^2^ = 99%) and was not statistically significant (*p* = 0.09). The 6-month protocol maintained a significant benefit compared with WMT (*p* = 0.008) (Supplementary Figure 7).

#### 3.4.4 CDR

Three trials assessed CDR scores (29, 36, 39). In the comparison of KTBAT combined with WMT, one study was included. The results indicated that KTBAT as an adjuvant therapy was associated with lower CDR scores than WMT alone (SMD = −0.87, 95% CI: [−1.26, −0.47], *p* < 0.0001). For KTBAT monotherapy versus WMT, two studies involving 272 participants were analyzed. The fixed-effect model analysis revealed no statistical heterogeneity (*I*^2^ = 0%, *p* = 0.89). KTBAT monotherapy was also more effective than WMT in improving clinical dementia ratings (SMD = −1.13, 95% CI: [−1.39, −0.88], *p* < 0.00001) (Figure 6).

**Figure 6:**
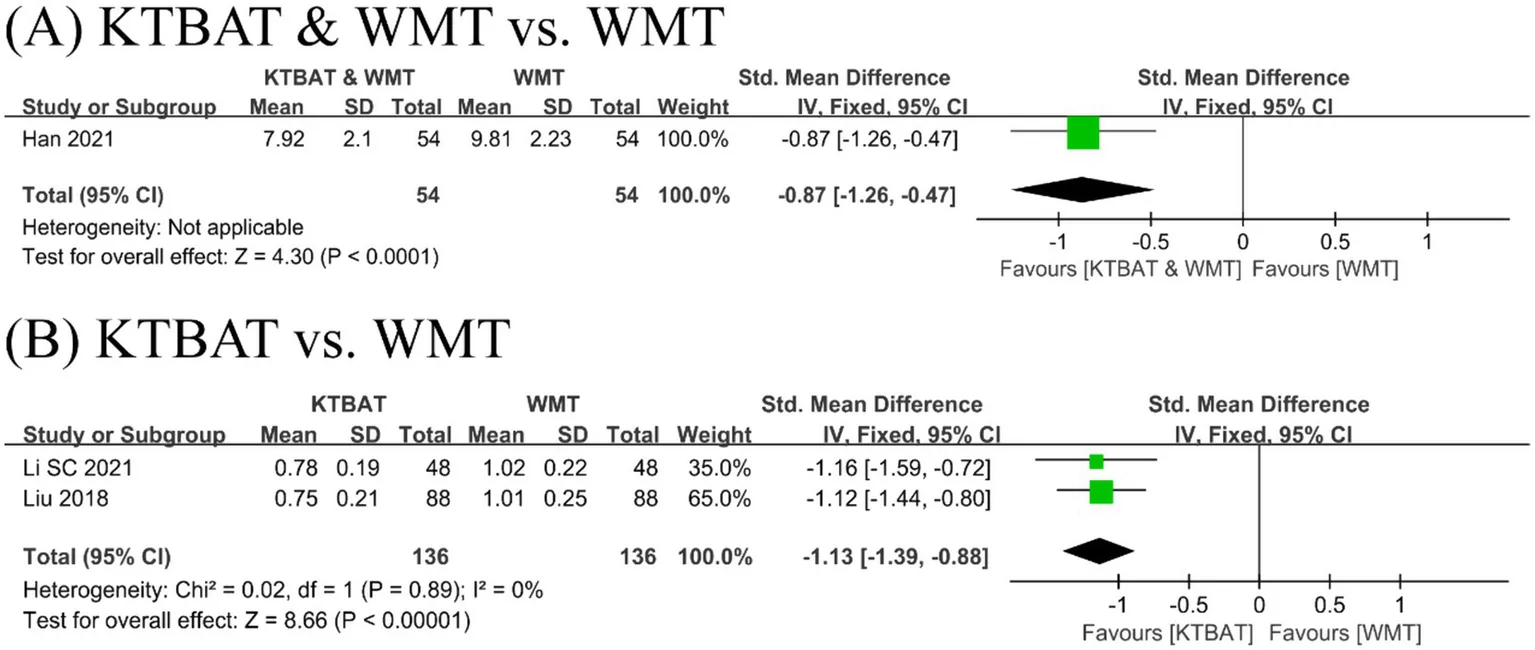
Forest plots of CDR comparing KTBAT with WMT in VaD treatments. **(A)** KTBAT combined with WMT for a subgroup efficacy analysis compared to using WMT alone. **(B)** A comparative analysis of the efficacy between KTBAT and WMT.

#### 3.4.5 BDNF

This section included six studies (33, 38, 46–48, 60). After excluding the crossover trial (48), five studies encompassing 437 patients were included in the quantitative synthesis. These analyses examined serum neurotrophic factor levels as potential mechanistic markers. In the three studies evaluating KTBAT combined with WMT, the pooled estimate suggested that KTBAT may be associated with higher BDNF levels (SMD = 1.02, 95% CI: [0.13, 1.90], *p* = 0.02) although heterogeneity was substantial (*I*^2^ = 91%). After excluding (47) in the sensitivity analysis, the heterogeneity was eliminated and the pooled effect remained significant (SMD = 0.55, 95% CI: [0.23, 0.86], *p* = 0.0006, *I*^2^ = 0%) (Supplementary Figure 8). In the monotherapy analysis of 2 studies with 173 participants, the pooled result failed to reach statistical significance (SMD = 1.20, 95% CI: [−0.08, 2.48], *p* = 0.07). (Figure 7).

**Figure 7:**
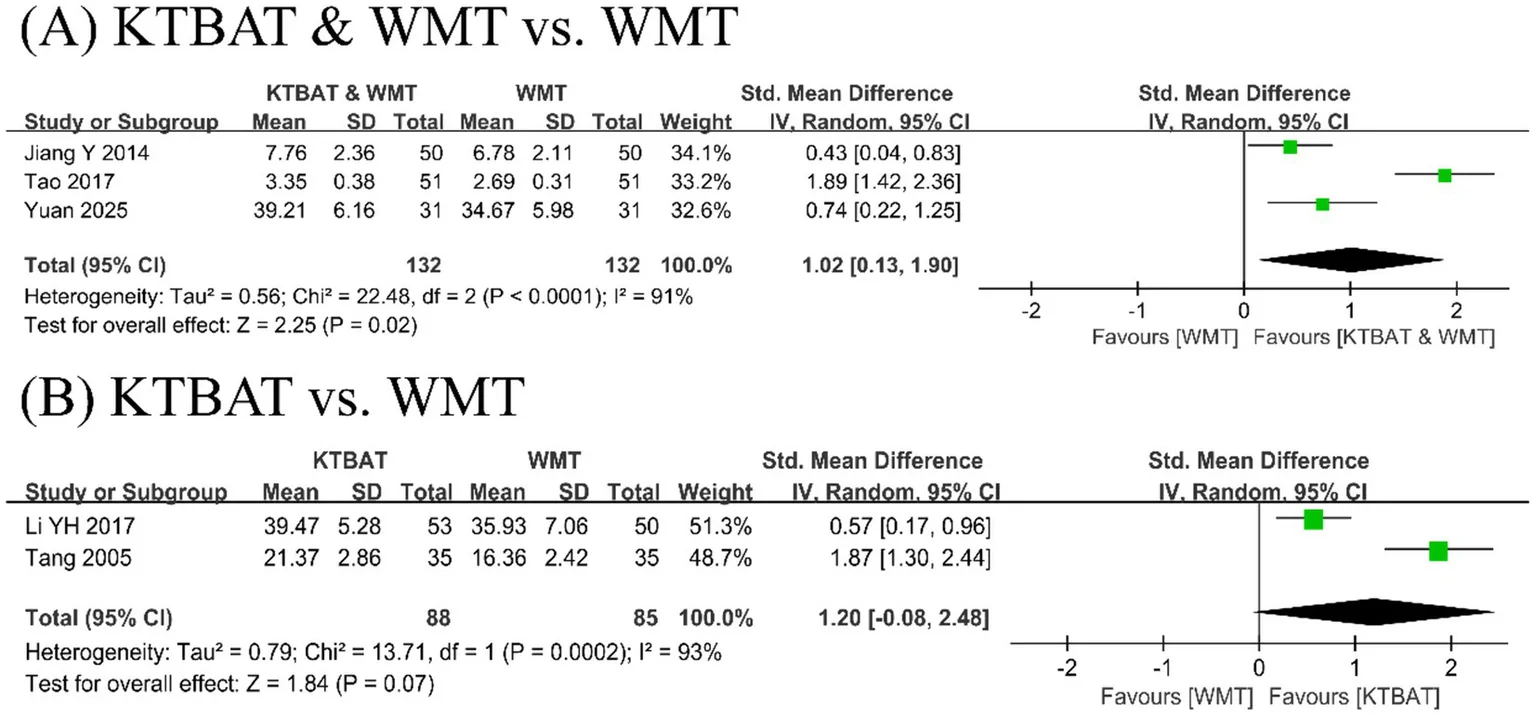
Forest plots of BDNF comparing KTBAT with WMT in VaD treatments. **(A)** KTBAT combined with WMT for a subgroup efficacy analysis compared to using WMT alone. **(B)** A comparative analysis of the efficacy between KTBAT and WMT.

#### 3.4.6 NGF

Five RCTs reported NGF levels (29, 32, 40, 50, 60). These studies, involving 469 participants, were stratified into three subgroups according to measurement units (Figure 8). In the μg/L subgroup, which included two studies, KTBAT combined with WMT was associated with significantly higher NGF levels (SMD = 1.01, 95% CI: [0.51, 1.50], *p* < 0.0001). The ng/L subgroup, based on one study, showed a similar result (SMD = 1.03, 95% CI: [0.63, 1.44], *p* < 0.00001). However, the ng/mL subgroup which integrated two studies failed to show a statistically significant difference (SMD = 0.23, 95% CI: [−0.04, 0.51], *p* = 0.10). When all studies were pooled, the overall estimate still favored KTBAT combined with WMT (SMD = 0.69, 95% CI: [0.27, 1.12], *p* = 0.001).

**Figure 8:**
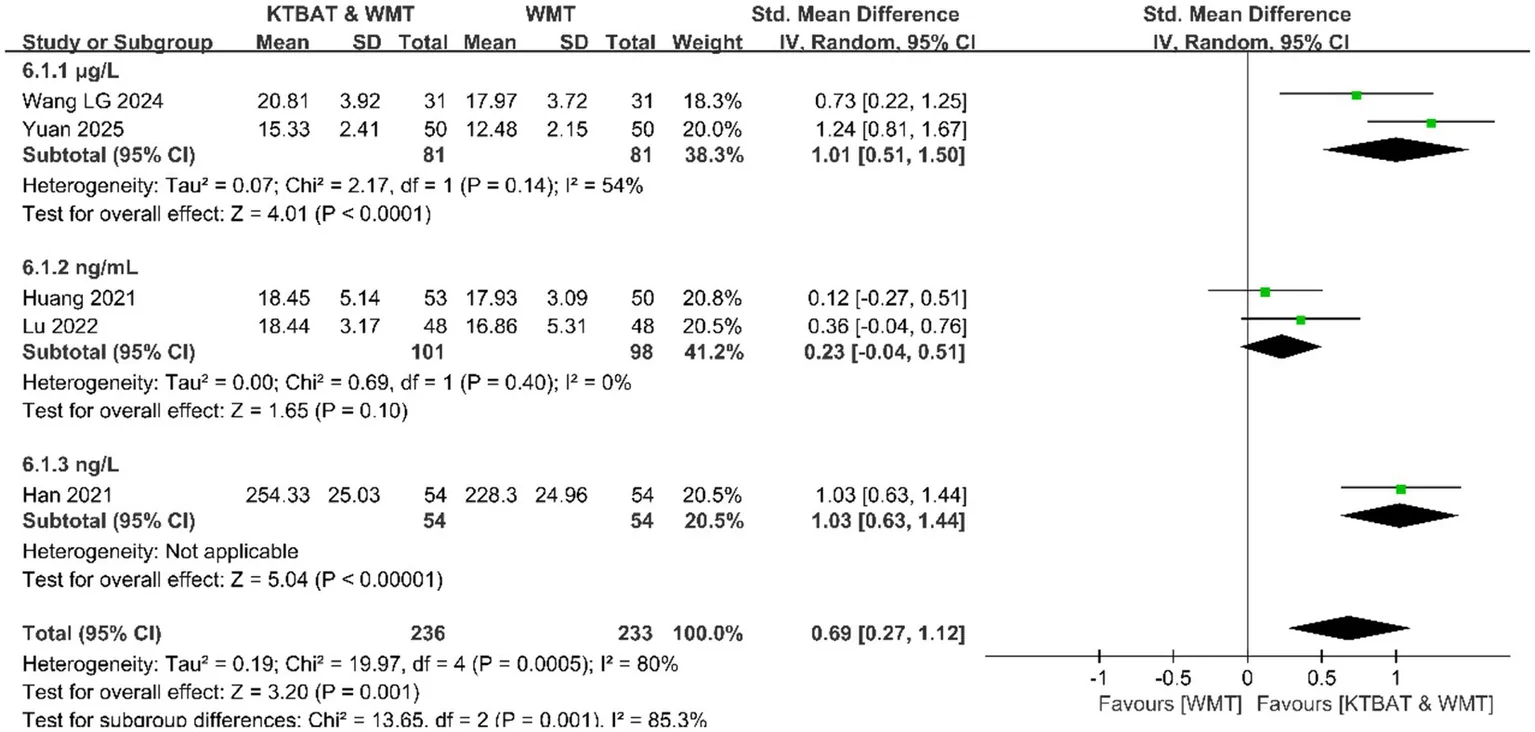
Forest plot of NGF comparing KTBAT plus WMT with WMT in VaD treatments.

#### 3.4.7 GDNF

Only one study reported the GDNF indicator (pg/mL) (29). This single trial involved 108 participants and compared KTBAT plus WMT with WMT monotherapy. The results showed that GDNF concentrations were significantly higher in the intervention group (MD = 56.56, 95% CI [44.84, 68.28], *p* < 0.00001) (Figure 9).

**Figure 9:**
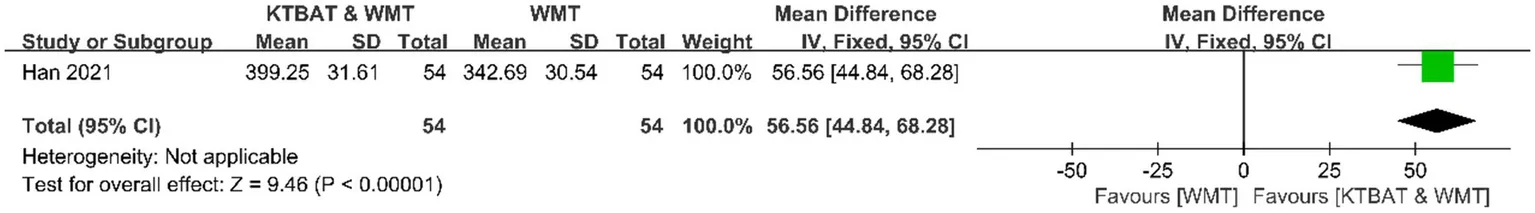
Forest plot of GDNF comparing KTBAT plus WMT with WMT in VaD treatments.

### 3.5 Adverse events

Among the 38 included studies, 9 trials (23.7%) reported AEs (28, 32, 35, 38, 48–50, 60, 63). However, three crossover trials reported only combined-period or non-poolable AE data and were not included in the quantitative AE synthesis. Thus, 6 trials (15.7%) were included in the pooled AE analysis, 8 trials (21.1%) explicitly stated that no AEs occurred, 3 trials (7.8%) reported AEs but were not pooled, and the remaining 21 trials (55.3%) did not mention AEs. None of the original studies discontinued treatment due to AEs. In the six trials reporting AEs, a total of 48 participants experienced AEs (22 in the KTBAT groups and 26 in the control groups). No serious AEs or deaths were reported. The reported AEs included insomnia, diarrhea, abdominal pain, dizziness, and rash. A summary of AEs reported in each trial is presented in Table 2.

**Table 2:** Adverse events of the included studies.

Included study	N at risk (T/C)	AEs in treatment group (specific manifestations, *n*)	AEs in control group (specific manifestations, *n*)	Any AE, *n* (%) (T/C)	Discontinuation due to AE (T/C)	AE reporting status
Guo et al. (28)	60/60	Rash (*n* = 2)	Rash (*n* = 2), elevated AST (*n* = 3)	2 (3.3%) / 5 (8.3%)	0/0	Reported (with AEs)
Huang (32)	53/50	Mild blood pressure drop (*n* = 4), nausea and vomiting (*n* = 2), decreased appetite (*n* = 1)	Dizziness (*n* = 2), mild blood pressure drop (*n* = 3), nausea (*n* = 1)	7 (13.2%) / 6 (12.0%)	0/0	Reported (with AEs)
Li (38)	119/119	Nausea, decreased appetite (*n* = 2)	Dizziness (*n* = 2), gastrointestinal discomfort (*n* = 6)	2 (1.7%) / 8 (6.7%)	0/0	Reported (with AEs)
Wang et al. (50)	31/31	Mild frequent urination (*n* = 2), lower abdominal pain (*n* = 1), rash (*n* = 2)	Mild frequent urination (*n* = 2), dizziness and nausea (*n* = 3), rash (*n* = 1)	5 (16.1%) / 6 (19.3%)	0/0	Reported (with AEs)
Yuan et al. (90)	50/50	Insomnia (*n* = 1), nausea and vomiting (*n* = 1), diarrhea (*n* = 2)	Explicitly stated no AEs	4 (8%) / 0	0/0	Reported (with AEs)
Zhong et al. (63)	35/35	Diarrhea (*n* = 1), nausea (*n* = 1)	Dizziness (*n* = 1)	2 (5.7%) / 1 (2.8%)	0/0	Reported (with AEs)
Jin et al. (35)	72/72	Dry mouth and sore throat (*n* = 2)		Not pooled	Not pooled	Not pooled
Wang et al. (48)	18/18	Dry mouth and sore throat (*n* = 2)		Not pooled	Not pooled	Not pooled
Wang et al. (49)	36/36	Dry mouth and sore throat (*n* = 4)		Not pooled	Not pooled	Not pooled
Dou and Gao (27)	47/47	NR	NR	NR	0/0	Not mentioned
Han et al. (29)	54/54	NR	NR	NR	0/0	Not mentioned
Hu et al. (30)	50/50	NR	NR	NR	0/0	Not mentioned
Hu and Yu (31)	37/30	NR	NR	NR	0/0	Not mentioned
Jiang and Xu (33)	34/32	NR	NR	NR	0/0	Not mentioned
Jiang (34)	30/30	NR	NR	NR	0/0	Not mentioned
Li (36)	48/48	0	0	0	0/0	Explicitly stated no AEs
Li et al. (37)	57/53	0	0	0	0/0	Explicitly stated no AEs
Liu (39)	88/88	NR	NR	NR	0/0	Not mentioned
Lu et al. (40)	48/48	NR	NR	NR	0/0	Not mentioned
Ma et al. (41)	46/46	NR	NR	NR	0/0	Not mentioned
Man et al. (42)	47/47	0	0	0	0/0	Explicitly stated no AEs
Peng and Dong (43)	28/28	0	0	0	0/0	Explicitly stated no AEs
Shao (44)	31/24	NR	NR	NR	0/0	Not mentioned
Sheng et al. (45)	45/45	0	0	0	0/0	Explicitly stated no AEs
Tang et al. (46)	40/40	0	0	0	0/0	Explicitly stated no AEs
Tao et al. (47)	46/46	NR	NR	NR	0/0	Not mentioned
Wang (51)	30/30	0	0	0	0/0	Explicitly stated no AEs
Wang (52)	38/38	NR	NR	NR	0/0	Not mentioned
Wu et al. (53)	32/32	NR	NR	NR	0/0	Not mentioned
Wu (54)	50/50	NR	NR	NR	0/0	Not mentioned
Yang et al. (55)	38/37	0	0	0	0/0	Explicitly stated no AEs
Yang et al. (56)	42/40	NR	NR	NR	0/0	Not mentioned
Yao (57)	51/51	NR	NR	NR	0/0	Not mentioned
Yao and Yang (58)	39/39	NR	NR	NR	0/0	Not mentioned
Yu (59)	44/44	NR	NR	NR	0/0	Not mentioned
Zhao (61)	49/49	NR	NR	NR	0/0	Not mentioned
Zheng et al. (62))	27/26	NR	NR	NR	0/0	Not mentioned
Zhu (64)	65/65	NR	NR	NR	0/0	Not mentioned

AST, aspartate aminotransferase; BP, blood pressure; C, control group; GI, gastrointestinal; NR, not reported; T, treatment group.

*N* at risk refers to the safety analysis population (participants who were randomized and received at least one dose of the allocated intervention). For trials reporting dropouts or exclusions before treatment initiation, *N* at risk = randomized number − number not receiving treatment. Percentages are calculated using N at risk as the denominator. “Reported (with AEs)” indicates that the trial provided adverse event data. “Explicitly stated no AEs” indicates that the trial actively stated that no AEs occurred (marked as 0). “Not mentioned” indicates that the trial did not mention AEs at all; this does not indicate the absence of AEs. “NR” indicates not reported. Enter “0” only when the original trial explicitly reports no AEs or no discontinuations due to AEs. Trials with dropouts should also be recorded as 0 if they clearly state that the dropouts were not due to AEs (e.g., participants were excluded before treatment at their own request). For crossover trials, only first-period data were eligible for pooling; combined-period or per-period repeated-measures data were not pooled. A total of 6 of 38 trials (15.7%) reported AEs, 8 trials (21.1%) explicitly stated no AEs, 3 trials (7.8%) did not pooled AEs and 21 trials (55.3%) did not mention AEs. No serious AEs or treatment-related deaths were reported in any included trial. All outcome measures related to the original study were complete, and none of the participants discontinued treatment due to AEs.

Three clinical trials reported safety outcomes for KTBAT plus WMT compared with WMT. No significant difference in AEs was observed between the two groups (*I*^2^ = 21%, RR = 0.85, 95% CI: [0.43, 1.68], *p* = 0.64). KTBAT monotherapy also showed comparable AEs rates to WMT (RR = 0.71, 95% CI: [0.32, 1.57], *p* = 0.40). (Figure 10). Sensitivity analyses, performed by removing each study in turn, did not materially change the results.

**Figure 10:**
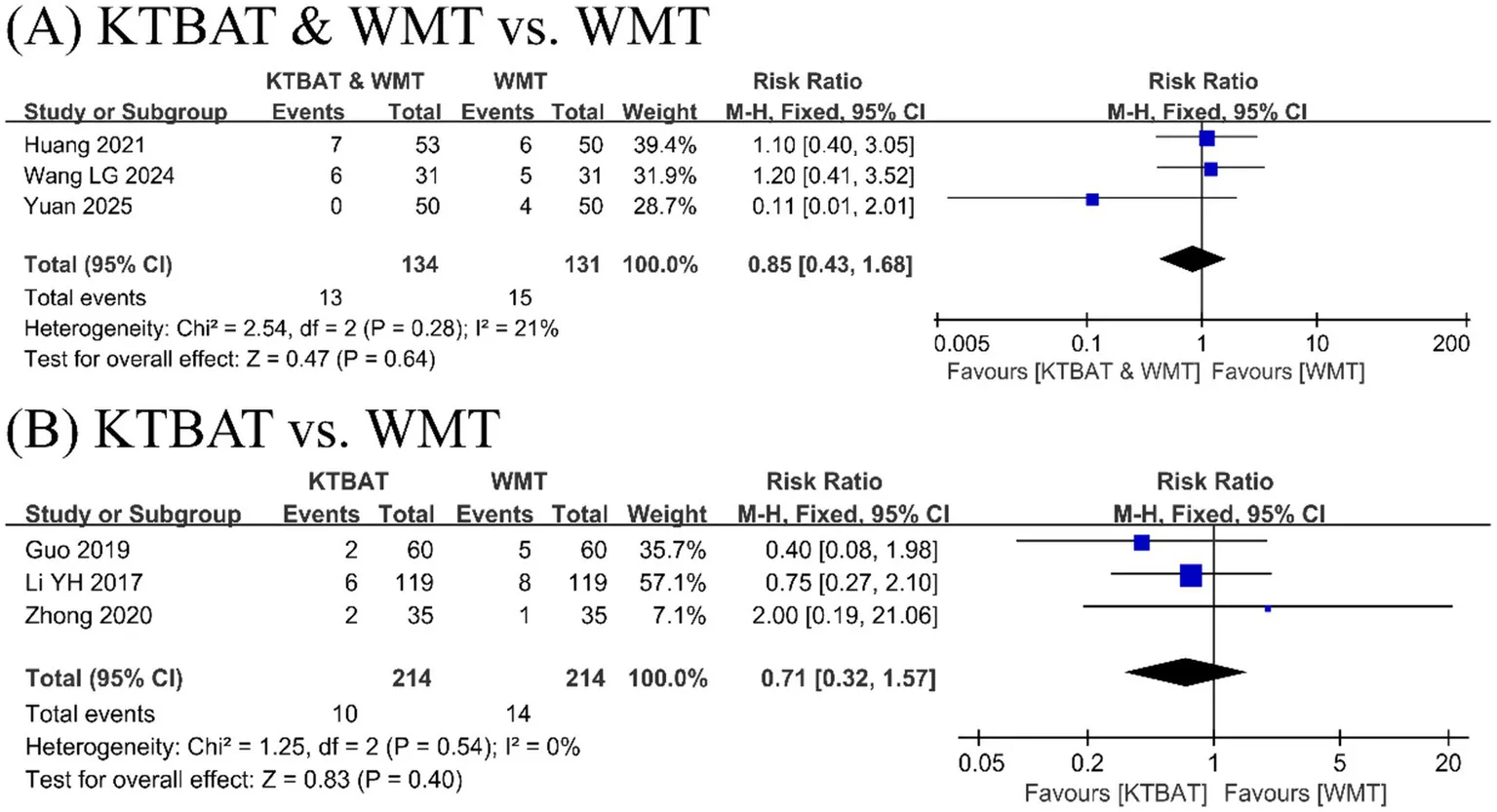
Forest plots of AEs comparing KTBAT with WMT in VaD treatments. **(A)** KTBAT combined with WMT for a subgroup efficacy analysis compared to using WMT alone. **(B)** A comparative analysis of the efficacy between KTBAT and WMT.

### 3.6 Sensitivity analysis and publication bias

To evaluate the robustness of the pooled estimates, leave-one-out sensitivity analyses were performed for the primary outcomes. For the clinical effectiveness rate, both the KTBAT plus WMT group (22 studies) and the KTBAT monotherapy group (13 studies) showed consistent results, with no notable change in the pooled RRs after omitting any individual study. These results suggest that the findings for clinical efficacy were stable. However, funnel plots inspection revealed asymmetry in both groups, indicating a possible publication bias in the reporting of clinical effectiveness rate. Sensitivity analyses for MMSE scores were conducted in the KTBAT plus WMT group (17 studies) and the KTBAT monotherapy group (13 studies). Although the initial pooled analyses showed moderate-to-high heterogeneity, the leave-one-out analyses yielded stable results, and no single study had a substantial influence on the pooled MD. The funnel plots for MMSE scores in both groups were also asymmetrical (Figure 11), suggesting a potential small-study effect or selective reporting. Overall, despite the potential publication bias, the main findings on the effects of KTBAT on VaD remained robust across sensitivity analyses.

**Figure 11:**
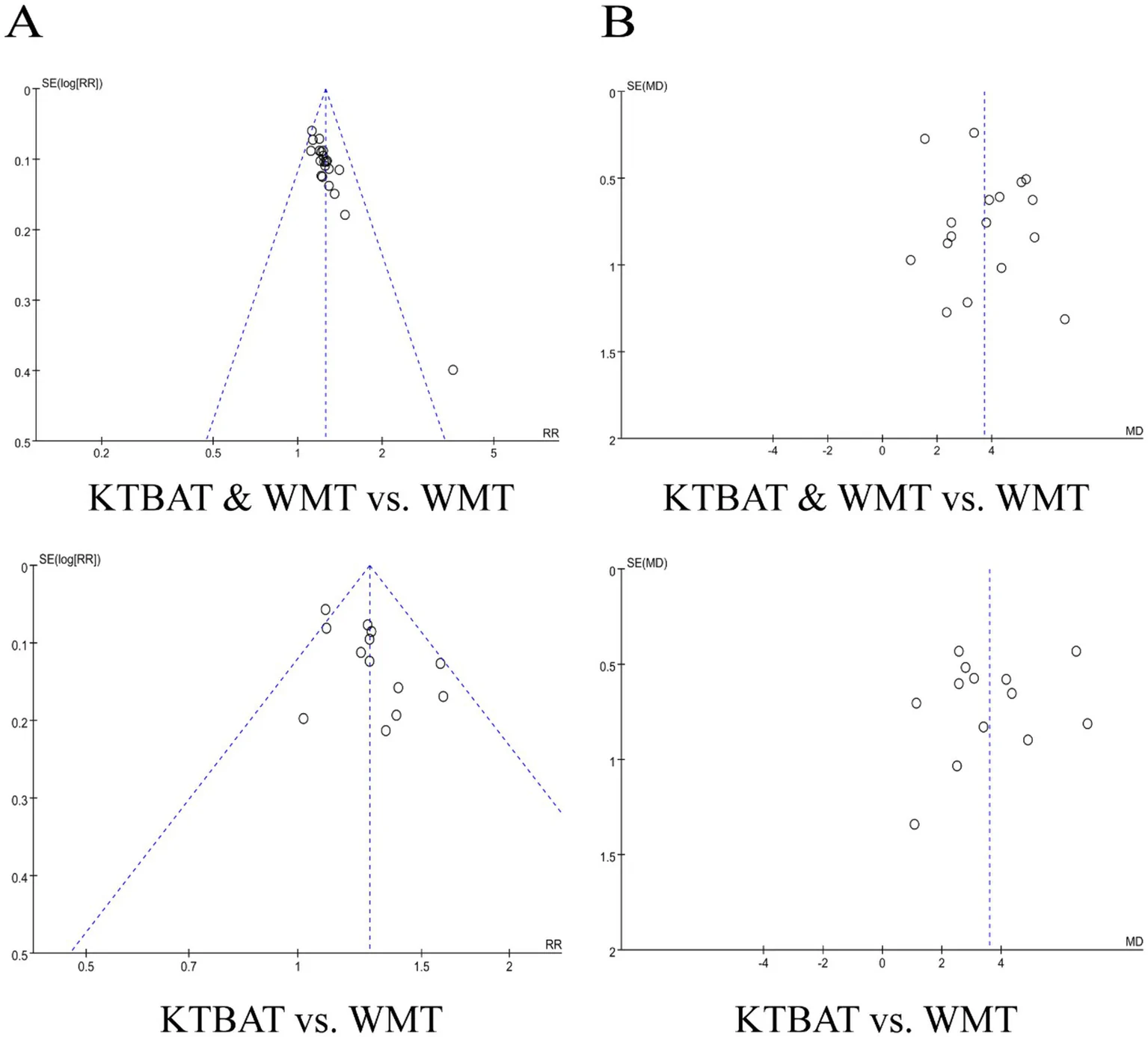
Funnel plots of clinical effectiveness rate and MMSE comparing KTBAT plus WMT or KTBAT alone with WMT in VaD treatments. **(A)** KTBAT combined with WMT for a subgroup efficacy analysis compared to using WMT alone. **(B)** A comparative analysis of the efficacy between KTBAT and WMT.

## 4 Discussion

### 4.1 Primary efficacy and safety outcomes

This systematic review analyzed 38 RCTs involving 3,357 participants to evaluate the efficacy and safety of KTBAT for VaD. To our knowledge, this is the first comprehensive meta-analysis to assess KTBAT for VaD. Our findings suggest that KTBAT, particularly when combined with WMT, was associated with improved clinical effectiveness and cognitive performance. In the combination therapy subgroup, MMSE and HDS scores showed greater improvements than WMT monotherapy. Although heterogeneity was observed in some cognitive outcomes, the overall direction of effect across the included studies was consistent, which supports the potential clinical value of KTBAT in different practice settings. However, it should be emphasized that these outcomes reflect symptomatic and cognitive improvement over a short treatment period, and they do not demonstrate that KTBAT modifies the underlying disease course of VaD. Importantly, statistically significant changes in serum biomarkers do not establish clinical benefit. Biomarkers are intermediate measures and are not surrogate endpoints for cognitive improvement or disease modification.

The observed clinical benefits of KTBAT may be partly explained by its multi-target and multi-level therapeutic effects. Pooled analyses suggested that KTBAT may be associated with higher serum BDNF, NGF, and GDNF levels. These neurotrophic factors are closely related to neuronal survival and synaptic plasticity. The observed changes may reflect a possible modulation of neurotrophic support, but the short-term nature of the included trials and the small number of studies mean that these findings do not establish that KTBAT attenuates neurological deficit or modifies the disease course.

Regarding safety, our results indicate that KTBAT does not significantly increase AEs compared with WMT. However, only six trials were included in the quantitative AE synthesis; three crossover trials reported AEs but were not pooled. The placebo-controlled evidence was limited and not quantitatively synthesised; therefore, the safety profile of KTBAT remains uncertain. In addition, the findings from the monotherapy analysis showed that KTBAT had comparable efficacy to conventional cognitive enhancers. This indicates that KTBAT may serve as an alternative therapeutic option for patients who cannot tolerate standard WMT.

### 4.2 Subgroup findings and heterogeneity sources

Substantial heterogeneity was observed in the improvement of cognitive scales, particularly MMSE and HDS. To explore potential sources of this variability, we conducted stratified analyses based on dosage forms and treatment duration. The subgroup analysis of MMSE suggested that dosage forms may have influenced the observed clinical outcomes. In the combination therapy subgroup, capsules and decoctions were consistently associated with greater improvements than WMT, whereas granules did not show statistically significant differences. This pattern may be partly related to differences in the bioavailability of active components among formulations. It also suggests that traditional decoctions and modern capsules may provide more stable exposure of neuroprotective compounds.

Regarding treatment duration, cognitive improvements were generally observed across interventions lasting from 2 to 6 months. Notably, the 6-month subgroup provided the most consistent evidence of benefit in both monotherapy and combination therapy. This may indicate that KTBAT has a relatively sustained effect on cognitive outcomes in patients with VaD. By contrast, the 1-month subgroup showed larger mean differences in some trials, which may reflect early treatment effects or differences in study characteristics. However, other clinical factors did not fully account for the remaining heterogeneity. Differences in baseline dementia severity and individualized herb dosages may have contributed to the residual variance.

Methodological differences may provide another explanation for the heterogeneity. Variations in assessor training and in the administration of cognitive scales could have introduced measurement bias across study centers. Similarly, heterogeneity in BDNF and NGF assay sensitivity and reporting units may have affected the comparability of biomarker results. The lack of standardized herbal processing methods may also have led to differences in active ingredient content. In addition, the absence of stratification by VaD subtype, such as subcortical ischemic vascular dementia and multi-infarct dementia (65), during patient enrollment may also have contributed to the observed heterogeneity. Unfortunately, the limited reporting of these details prevented further meta-regression analyses. Nevertheless, the overall direction of effect sizes remained consistent across most subgroups, which supports the potential therapeutic value of KTBAT. Future studies should adopt more standardized protocols to reduce clinical and methodological heterogeneity.

### 4.3 Pathophysiology of VaD and current treatments

VaD is a neurodegenerative disease that results from various cerebrovascular pathologies. Its pathogenesis involves multiple mechanisms, including apoptosis, inflammatory responses, and oxidative stress (66, 67). In addition, stroke, hypertension-related cerebral small vessel disease, and chronic cerebral hypoperfusion (CCH) are regarded as major contributors to cognitive decline in patients with VaD (68, 69). CCH can induce mitochondrial dysfunction and promote excessive reactive oxygen species (ROS) generation, thereby worsening blood–brain barrier (BBB) disruption, neuroinflammatory responses, and neuronal apoptosis, which may ultimately contribute to cognitive deterioration (70).

Currently, no drugs have been approved by the Food and Drug Administration (FDA) specifically for the treatment of VaD (5). As a result, medications approved for AD are often used in clinical practice to alleviate vascular cognitive impairment (71). Donepezil, galantamine, and memantine have been used in multiple regions to improve cognitive function in patients with VaD (72, 73). In light of the complex pathophysiology of VaD, the multi-target and multi-pathway strategies of TCM may offer potential for managing symptoms and cognitive impairment; however, disease-modifying effects remain unproven (74, 75). Diverse components derived from CHMs could ameliorate the neurovascular glial unit dysfunction in VaD (76, 77).

Additionally, some of the included trials used traditional Chinese medicine syndrome scores to assess clinical efficacy, suggesting that traditional Chinese medicine may help alleviate the accompanying symptoms of VaD, such as soreness in the lower back and knees and weakness. From a mechanistic perspective, BDNF is a key regulator of synaptic plasticity. Therefore, the relevant statistical findings in this study regarding KTBAT and neurotrophic factors may indicate that its mechanism of action is associated with synaptic remodeling and neuronal protection. It should be considered a hypothesis for future mechanistic research rather than evidence of disease modification.

### 4.4 TCM theoretical framework for VaD

According to the theory of TCM, the brain is regarded as the “sea of marrow” (Nao Wei Sui Hai), whose functional state is intrinsically linked to the abundance of kidney essence (Shen Jing). Ancient TCM literature also describes “post-stroke amnesia” (Zhong Feng Hou Shan Wang), highlighting the recognized connection between cerebrovascular events and cognitive impairment. Within this theoretical framework, the etiology and pathogenesis of VaD are primarily attributed to kidney deficiency. Specifically, the proper functioning of the brain collaterals (Nao Luo) depends on nourishment from kidney essence; deficiency of this essence leads to an insufficient “sea of marrow,” resulting in inadequate nourishment of the brain. Additionally, Qi deficiency can impair the propulsion of blood, leading to blood stasis, which further obstructs the brain collaterals and exacerbates neurological damage. Kidney deficiency and blood stasis are also considered to be mutually reinforcing and can exacerbate each other in a vicious cycle. Therefore, “kidney deficiency and blood stasis” is considered an important pathological mechanism of VaD. Accordingly, the therapeutic principle of tonifying kidney essence and activating blood circulation to resolve stasis constitutes a common treatment approach for this condition. This principle is embodied in numerous classic herbal formulas, such as Dihuang Yinzi, Bushen Huoxue Tang, and Yisui Zengzhi Decoction.

### 4.5 Pharmacological mechanisms of key CHMs

Based on the herbs most frequently used in KTBAT (Supplementary Table 2), we reviewed and summarized the relevant preclinical studies, briefly outlining their TCM effects and pharmacological mechanisms in relation to VaD.

Among the blood-activating herbs, *Chuanxiong Rhizoma* (76, 78, 79), *Salviae Miltiorrhizae Radix et Rhizoma* (80, 81), and *Carthami Flos* (82) may act primarily by improving hemodynamics, inhibiting thrombosis, and preserving endothelial integrity. In contrast, *Paeoniae Radix Rubra* (83, 84) predominantly contributes to the restoration of mitochondrial function, alongside the suppression of neuroinflammation and oxidative stress. Furthermore, *Angelicae Sinensis Radix* exhibits neuroprotective properties via cholinesterase inhibition (85, 86), which could potentially influence the clinical progression of AD and VaD (87, 88). In clinical practice, these herbs are often combined in paired formulations, such as *Chuanxiong Rhizoma* coupled with *Angelicae Sinensis Radix*, as well as *Salviae Miltiorrhizae Radix et Rhizoma* paired with *Carthami Flos,* in order to enhance their blood-activating effects.

Regarding kidney-tonifying ingredients, *Polygoni Multiflori Radix Praeparata* (89), *Polygonati Rhizoma* (90), and *Schisandrae Chinensis Fructus* (91, 92), may exert pharmacological effects mainly by reducing oxidative damage, attenuating neuronal apoptosis, and modulating microglia-mediated inflammatory responses. Similarly functioning as a classical botanical pair, *Cistanches Herba* (93, 94) and *Epimedii Herba* (95, 96) demonstrate a combined capacity to inhibit cellular apoptosis, modulate neurotransmitter levels for cholinergic neuroprotection, and subsequently ameliorate blood–brain barrier dysfunction. Additionally, *Cornel iridoid glycoside* (CIG) has been reported to modulate neurotrophic factor expression within the cortex and hippocampus of rats subjected to chronic cerebral ischemia (97), while simultaneously downregulating microglia-driven inflammation (98).

Collectively, the therapeutic effects of KTBAT in VaD are unlikely to depend on a single pathway. Instead, they may reflect a coordinated biological response involving both improved cerebral perfusion through blood-activating herbs and enhanced neuronal resilience through kidney-tonifying herbs. However, these mechanistic interpretations are largely based on preclinical evidence and indirect clinical markers. Clinically, these mechanisms may inspire future multi-target research, but they require confirmation in long-term, adequately powered trials.

## 5 Limitations

This review has several limitations. First, the overall methodological quality of the included RCTs was relatively low, as the majority of studies lacked detailed descriptions of blinding procedures, rendering them susceptible to performance and detection biases. Second, because all of the included studies were conducted in China, the generalizability of the findings to other populations may be limited. Third, the included studies predominantly consisted of single-center trials with small sample sizes, which may limit statistical power. Fourth, long-term follow-up data were generally lacking across these trials, precluding a definitive assessment of the therapy’s long-term efficacy and safety. Neurotrophic factor outcomes were reported in only a small subset of trials, and the available short-term data do not establish disease-modifying effects. Fifth, three crossover trials could not be included in the quantitative synthesis because only combined-period or non-poolable data were reported; this may have limited the assessment of safety and placebo-controlled effects. Additionally, although comprehensive subgroup analyses were performed for outcomes with substantial heterogeneity, some residual heterogeneity remained unexplained. Finally, the variations in herbal formulations and concurrent WMT regimens complicated the interpretation of the true treatment effect.

## 6 Conclusion

This study provides modern evidence-based support for the TCM theory of “kidney deficiency and blood stasis” in VaD. Current evidence suggests that KTBAT, as an adjunct therapy or monotherapy, may improve clinical effectiveness and cognitive function in patients with VaD; however, its safety profile is not well established due to limited AE reporting. Given the suboptimal methodological quality and inherent heterogeneity of the included studies, these findings warrant further confirmation through large-scale, high-quality RCTs. Future clinical investigations should prioritize multi-center, randomized, double-blind, placebo-controlled designs with standardized outcome measures and extended follow-up durations. Moreover, we recommend incorporating TCM syndrome scales to precisely stratify specific populations with the “Kidney Deficiency and Blood Stasis” syndrome, thereby optimizing syndrome-prescription correspondence. Concurrently, advanced technologies such as network pharmacology and molecular biology could be used to further elucidate the underlying pharmacological mechanisms and critical target pathways of KTBAT in combating dementia.

## Data Availability

The datasets presented in this study can be found in online repositories. The names of the repository/repositories and accession number(s) can be found in the article/Supplementary material.
