# Supplementary material for: Efficacy and safety of traditional Chinese medicine kidney-tonifying and blood-activating therapy in the treatment of vascular dementia: a systematic review and meta-analysis
Source: Front Neurol. 2026 Oct 20;17:1901245. doi: 10.3389/fneur.2026.1901245 (PMC13729250; doi:10.3389/fneur.2026.1901245)
Supplement: Supplementary file 1 [file Supplementary_file_1.docx]

Supplementary Material

# 1 Supplementary Text

**PubMed:**

For retrieval of databases, the keywords of search are the same, with taking PubMed as an example:

#1 ("Dementia, Vascular"[Mesh]) OR (((((((((((((((((((((((((((((((((((((((Dementias, Vascular[Title/Abstract]) OR (Vascular Dementias[Title/Abstract])) OR (Vascular Dementia[Title/Abstract])) OR (Arteriosclerotic Dementia[Title/Abstract])) OR (Arteriosclerotic Dementias[Title/Abstract])) OR (Dementia, Arteriosclerotic[Title/Abstract])) OR (Dementias, Arteriosclerotic[Title/Abstract])) OR (Binswanger Disease[Title/Abstract])) OR (Disease, Binswanger[Title/Abstract])) OR (Encephalopathy, Binswanger[Title/Abstract])) OR (Chronic Progressive Subcortical Encephalopathy[Title/Abstract])) OR (Binswanger Encephalopathy[Title/Abstract])) OR (Leukoencephalopathy, Subcortical[Title/Abstract])) OR (Leukoencephalopathies, Subcortical[Title/Abstract])) OR (Subcortical Leukoencephalopathies[Title/Abstract])) OR (Encephalopathy, Subcortical Arteriosclerotic[Title/Abstract])) OR (Encephalopathy, Chronic Progressive Subcortical[Title/Abstract])) OR (Encephalopathy, Subcortical, Chronic Progressive[Title/Abstract])) OR (Subcortical Encephalopathy, Chronic Progressive[Title/Abstract])) OR (Subcortical Leukoencephalopathy[Title/Abstract])) OR (Subcortical Arteriosclerotic Encephalopathy[Title/Abstract])) OR (Arteriosclerotic Encephalopathy, Subcortical[Title/Abstract])) OR (Arteriosclerotic Encephalopathies, Subcortical[Title/Abstract])) OR (Encephalopathies, Subcortical Arteriosclerotic[Title/Abstract])) OR (Subcortical Arteriosclerotic Encephalopathies[Title/Abstract])) OR (Encephalopathy, Binswanger's[Title/Abstract])) OR (Binswanger's Encephalopathy[Title/Abstract])) OR (Encephalopathy, Binswangers[Title/Abstract])) OR (Binswanger's Disease[Title/Abstract])) OR (Binswangers Disease[Title/Abstract])) OR (Disease, Binswanger's[Title/Abstract])) OR (Subcortical Vascular Dementia[Title/Abstract])) OR (Dementias, Subcortical Vascular[Title/Abstract])) OR (Dementia, Subcortical Vascular[Title/Abstract])) OR (Subcortical Vascular Dementias[Title/Abstract])) OR (Vascular Dementias, Subcortical[Title/Abstract])) OR (Vascular Dementia, Subcortical[Title/Abstract])) OR (Vascular Dementia, Acute Onset[Title/Abstract])) OR (Acute Onset Vascular Dementia[Title/Abstract]))

#2: ((((((((((((((((((((((((((((((((((("Kidney deficiency and blood stasis"[Title/Abstract]) OR (Shenxu Xueyu[Title/Abstract])) OR ("Kidney tonifying and blood activating therapy"[Title/Abstract])) OR ("kidney-tonifying and blood-activating"[Title/Abstract])) OR ("Tonifying Kidney and Invigorating Blood Circulation"[Title/Abstract])) OR ("Tonifying kidney and activating blood circulation"[Title/Abstract])) OR ("Tonifying Kidney and Activating Blood Prescription"[Title/Abstract])) OR ("Tonifying Kidney and Activating Blood"[Title/Abstract])) OR ("Tonifying Kidney and Activating Blood Granules"[Title/Abstract])) OR (Bushen Huoxue formula[Title/Abstract])) OR (Bushen Huoxue Recipe[Title/Abstract])) OR (Bushen Huoxue Prescription[Title/Abstract])) OR (Bushen Huoxue herbal medicine[Title/Abstract])) OR (Bushen Huoxue[Title/Abstract])) OR (Bushen Huoxue traditional Chinese medicine[Title/Abstract])) OR (Bushen Huoxue Granules[Title/Abstract])) OR (Buyang Huanwu Tang[Title/Abstract])) OR (Buyang Huanwu[Title/Abstract])) OR (Dihuang Yinzi[Title/Abstract])) OR (Rehmannia Decoction[Title/Abstract])) OR (Gunao Yizhi Tang[Title/Abstract])) OR (Gunao Yizhi[Title/Abstract])) OR (Yizhi Tongmai Formula[Title/Abstract])) OR (Yizhi Tongmai[Title/Abstract])) OR (Tongqiao Huoxue Decoction[Title/Abstract])) OR (Tongqiao Huoxue[Title/Abstract])) OR ("Kidney tonifying and stasis resolving formula"[Title/Abstract])) OR (Bushen Huayu formula[Title/Abstract])) OR (Bushen Huayu[Title/Abstract])) OR (Yisui Zengzhi Tang[Title/Abstract])) OR (Yisui Zengzhi[Title/Abstract])) OR (Bushen Yizhi Decoction[Title/Abstract])) OR (Bushen Yizhi[Title/Abstract])) OR (Tongluo Yinao Pill[Title/Abstract])) OR (Tongluo Yinao[Title/Abstract])) OR (Naoxintong[Title/Abstract])

#1 AND #2

# 2 Supplementary Figures and Tables

## 2.1 Supplementary figures

Supplementary Figure 1 Subgroup of MMSE dosage forms.

**Supplementary Figure 2** Subgroup of MMSE treatment durations.

**Supplementary Figure 3** Subgroup of MMSE dosage forms.

Supplementary Figure 4 Subgroup of MMSE treatment durations.

**Supplementary Figure 5** Sensitivity Analysis of HDS.

Supplementary Figure 6 Subgroup of HDS dosage forms.

Supplementary Figure 7 Subgroup of HDS treatment durations.

Supplementary Figure 8 Sensitivity Analysis of BDNF.

## 2.2 Supplementary Tables

Supplementary Table 1 Literature Quality Assessment

| **Author** | **Random sequence generation** | **Allocation concealment** | **Blinding of participants and personnel** | **Blinding of outcome assessment** | **Incomplete outcome data** | **Selective reporting** | **Other bias** |
| --- | --- | --- | --- | --- | --- | --- | --- |
| Jiang Y 2014 | Low risk of bias | Unclear risk of bias | Unclear risk of bias | Unclear risk of bias | Low risk of bias | Low risk of bias | Unclear risk of bias |
| Yuan 2025 | Low risk of bias | Unclear risk of bias | Unclear risk of bias | Unclear risk of bias | Low risk of bias | Low risk of bias | Unclear risk of bias |
| Wang LG 2024 | Low risk of bias | Unclear risk of bias | Unclear risk of bias | Unclear risk of bias | Low risk of bias | Low risk of bias | Unclear risk of bias |
| Ma 2019 | Low risk of bias | Unclear risk of bias | Unclear risk of bias | Unclear risk of bias | Low risk of bias | Low risk of bias | Unclear risk of bias |
| Guo 2019 | Unclear risk of bias | Unclear risk of bias | Unclear risk of bias | Unclear risk of bias | Low risk of bias | Low risk of bias | Unclear risk of bias |
| Tang 2005 | Low risk of bias | Unclear risk of bias | Unclear risk of bias | Unclear risk of bias | Low risk of bias | Low risk of bias | Unclear risk of bias |
| Zhong 2020 | Low risk of bias | Unclear risk of bias | Unclear risk of bias | Unclear risk of bias | Low risk of bias | Low risk of bias | Unclear risk of bias |
| Zheng 2009 | Unclear risk of bias | Unclear risk of bias | Unclear risk of bias | Unclear risk of bias | Low risk of bias | Low risk of bias | Unclear risk of bias |
| Sheng 2009 | Unclear risk of bias | Unclear risk of bias | Unclear risk of bias | Unclear risk of bias | Low risk of bias | Unclear risk of bias | Unclear risk of bias |
| Jiang YY 2006 | Low risk of bias | Unclear risk of bias | Unclear risk of bias | Unclear risk of bias | Unclear risk of bias | High risk of bias | Unclear risk of bias |
| Wang FW 2005 | Unclear risk of bias | Unclear risk of bias | Low risk of bias | Low risk of bias | Low risk of bias | Unclear risk of bias | Unclear risk of bias |
| Wu QF 2021 | Low risk of bias | Unclear risk of bias | Unclear risk of bias | Unclear risk of bias | Low risk of bias | Low risk of bias | Unclear risk of bias |
| Wu LC 2025 | Low risk of bias | Unclear risk of bias | Unclear risk of bias | Unclear risk of bias | Low risk of bias | Low risk of bias | Unclear risk of bias |
| Li SG 2015 | Unclear risk of bias | Unclear risk of bias | Unclear risk of bias | Unclear risk of bias | Low risk of bias | Low risk of bias | Unclear risk of bias |
| Hu CC 2023 | Low risk of bias | Unclear risk of bias | Unclear risk of bias | Unclear risk of bias | Low risk of bias | Low risk of bias | Unclear risk of bias |
| Liu 2018 | Low risk of bias | Unclear risk of bias | Unclear risk of bias | Unclear risk of bias | Low risk of bias | Low risk of bias | Unclear risk of bias |
| Hu YN 2013 | Unclear risk of bias | Unclear risk of bias | Unclear risk of bias | Unclear risk of bias | Low risk of bias | Low risk of bias | Unclear risk of bias |
| Peng 2007 | Low risk of bias | Unclear risk of bias | Unclear risk of bias | Unclear risk of bias | Low risk of bias | Low risk of bias | Unclear risk of bias |
| Yang HW 2025 | Low risk of bias | Unclear risk of bias | Unclear risk of bias | Unclear risk of bias | Low risk of bias | Low risk of bias | Unclear risk of bias |
| Jin 2007 | Unclear risk of bias | Unclear risk of bias | Low risk of bias | Low risk of bias | Low risk of bias | Low risk of bias | Unclear risk of bias |
| Li YH 2017 | Unclear risk of bias | Unclear risk of bias | Unclear risk of bias | Unclear risk of bias | Low risk of bias | Low risk of bias | Unclear risk of bias |
| Huang 2021 | Low risk of bias | Unclear risk of bias | Unclear risk of bias | Unclear risk of bias | Low risk of bias | Low risk of bias | Unclear risk of bias |
| Lu 2022 | Low risk of bias | Unclear risk of bias | Unclear risk of bias | Unclear risk of bias | Low risk of bias | Low risk of bias | Unclear risk of bias |
| Dou 2023 | Unclear risk of bias | Unclear risk of bias | Unclear risk of bias | Unclear risk of bias | Low risk of bias | Low risk of bias | Unclear risk of bias |
| Yao YJ 2021 | Low risk of bias | Unclear risk of bias | Unclear risk of bias | Unclear risk of bias | Low risk of bias | Low risk of bias | Unclear risk of bias |
| Man 2019 | Low risk of bias | Unclear risk of bias | Unclear risk of bias | Unclear risk of bias | Low risk of bias | Low risk of bias | Unclear risk of bias |
| Zhu 2015 | Low risk of bias | Unclear risk of bias | Unclear risk of bias | Unclear risk of bias | Low risk of bias | Low risk of bias | Unclear risk of bias |
| Wang TS 2016 | Low risk of bias | Unclear risk of bias | Unclear risk of bias | Unclear risk of bias | Low risk of bias | Low risk of bias | Unclear risk of bias |
| Zhao 2024 | Low risk of bias | Unclear risk of bias | Unclear risk of bias | Unclear risk of bias | Low risk of bias | Low risk of bias | Unclear risk of bias |
| Yu 2018 | Unclear risk of bias | Unclear risk of bias | Unclear risk of bias | Unclear risk of bias | Low risk of bias | Low risk of bias | Unclear risk of bias |
| Wang SF 2015 | Unclear risk of bias | Unclear risk of bias | Unclear risk of bias | Unclear risk of bias | Low risk of bias | Low risk of bias | Unclear risk of bias |
| Shao 2003 | Unclear risk of bias | Unclear risk of bias | Unclear risk of bias | Unclear risk of bias | Low risk of bias | Low risk of bias | Unclear risk of bias |
| Yang P 2019 | Unclear risk of bias | Unclear risk of bias | Unclear risk of bias | Unclear risk of bias | Low risk of bias | Low risk of bias | Unclear risk of bias |
| Wang FW 2004 | Unclear risk of bias | Unclear risk of bias | Low risk of bias | Low risk of bias | Low risk of bias | Low risk of bias | Unclear risk of bias |
| Han 2021 | Low risk of bias | Unclear risk of bias | Unclear risk of bias | Unclear risk of bias | Low risk of bias | Low risk of bias | Unclear risk of bias |
| Tao 2017 | Unclear risk of bias | Unclear risk of bias | Unclear risk of bias | Unclear risk of bias | Low risk of bias | Low risk of bias | Unclear risk of bias |
| Yao H 2017 | Unclear risk of bias | Unclear risk of bias | Unclear risk of bias | Unclear risk of bias | Low risk of bias | Low risk of bias | Unclear risk of bias |
| Li SC 2021 | Low risk of bias | Unclear risk of bias | Unclear risk of bias | Unclear risk of bias | Low risk of bias | Low risk of bias | Unclear risk of bias |

Supplementary Table 2 Composition and Proportions of TCM Herbs for KTBAT.

| **Included study** | **Names of TCM decoctions** | **Dosage forms** | **TCM decoction formulas** | **Decoction Preparation and Administration Methods** | **The percentage of CHMs for KTBAT (%)** | **Ranking of Single-Herb Medicinal Use Frequency** |
| --- | --- | --- | --- | --- | --- | --- |
| Han 2021 | Yisui Zengzhi Decoction | Decoction | *Astragali Radix* 60g, *Ligustri Lucidi Fructus* 30g, *Ecliptae Herba* 20g, *Polygonati Rhizoma* 15g, *Achyranthis Bidentatae Radix* 15g, *Lycii Fructus* 15g*, Spatholobi Caulis* 15g, *Cuscutae Semen* 12g, *Chuanxiong Rhizoma* 12g*, Epimedii Herba* 9g, *Paeoniae Radix 9g, Persicae Semen* 9g, *Carthami Flos* 9g, *Foeniculi Fructus* 6g, *Notoginseng Radix et Rhizoma* 3g | Boil the medicinal herbs in water, then collect 500 mL of the decoction and take 250 mL orally in the morning and evening | 81.25% | Chuanxiong Rhizoma (20) |
| Ma 2019 | Bushen Huoxue Decoction | Decoction | *Astragali Radix* 20g, *Corni Fructus* 10g*, Polygoni Multiflori Radix* 15g, *Rehmanniae Radix Praeparata* 15g, *Dioscoreae Rhizoma* 15g, *Achyranthis Bidentatae Radix* 10g, *Eucommiae Cortex* 10g, *Polygonati Rhizoma* 10g, *Chaenomelis Fructus* 5g, *Paeoniae Radix Rubra* 10g, *Carthami Flos* 5g, *Chuanxiong Rhizoma* 12g, *Pheretima* 15g, *Persicae Semen* 10g, *Acori Tatarinowii Rhizoma* 10g, *Aucklandiae Radix* 10g | Take the clay pot to boil with strong fire first, then simmer with gentle fire. Among them, the first decoction is 20 minutes, the second decoction is 15 minutes. The decocted liquid is combined, taken warm in the morning and evening, one dose per day, 30 days for one course, a total of three courses. | 75.00% | Rehmanniae Radix Praeparata (12) |
| Li SG 2015 | Dihuang Yinzi Capsule | Capsule | *Rehmanniae Radix Praeparata* 60g, *Corni Fructus* 45g, *Morindae Officinalis Radix* 45g, *Cistanches Herba* 45g, *Dendrobii Caulis* 45g, *Ophiopogonis Radix* 45g, *Schisandrae Chinensis Fructus* 30g, *Aconiti Lateralis Radix Praeparata* 30g, *Cinnamomi Cortex* 30g, *Poria* 30g, *Acori Tatarinowii Rhizoma* 30g, *Polygalae Radix* 30g, *Menthae Haplocalycis Herba* 10g, *Hominis Placenta* 50g, *Notoginseng Radix et Rhizomac* 30g (Powdered product, capsule set, each capsule contains 0.4 g of crude drug.) | Take five tablets per dose, twice daily. Each course lasts 30 days, for a total of six courses. | 73.33% | Polygoni Multiflori Radix Praeparata (11) |
| Wang SF 2015 | Xinjia Dihuang Yinzi | Decoction | *Rehmanniae Radix Praeparata* 24g, *Morindae Officinalis Radix* 12g, *Corni Fructus* 15g, *Dendrobii Caulis* 15g, *Cistanches Herba* 15g, *Aconiti Lateralis Radix Praeparata* 6g, *Schisandrae Chinensis Fructus* 15g, *Cinnamomi Cortex* 10g, *Poria* 15g, *Ophiopogonis Radix* 15g, *Acori Tatarinowii Rhizoma* 15g, *Polygalae Radix* 6g, *Menthae Haplocalycis Herba* 6g, *Curcumae Radix* 10g, *Hirudo* 6g | Take one dose daily, divided into two doses taken on an empty stomach in the morning and evening. One course of treatment lasts 30 days. | 73.33% | Corni Fructus (11) |
| Yao H 2017 | Gunao Yizhi Tang | Decoction | *Rehmanniae Radix Praeparata* 20g, *Dioscoreae Rhizoma* 15g, *Lycii Fructus* 12g, *Rehmanniae Radix* 12g, *Corni Fructus* 12g, *Polygalae Radix* 10g, *Acori Tatarinowii Rhizoma* 10g, *Curcumae Radix* 9g, *Chuanxiong Rhizoma* 10g, *Salviae Miltiorrhizae Radix et Rhizoma* 12g, *Glycyrrhizae Radix et Rhizoma Praeparata cum Melle* 9g | Take one dose daily; boil in water as usual and take warm in the morning and evening. | 72.73% | *Alpiniae Oxyphyllae Fructus* (10) |
| Wang TS 2016 | Tongqiao Huoxue Decoction | Decoction | *Persicae Semen* 15g, *Chuanxiong Rhizoma* 15g, *Carthami Flos* 10g, *Paeoniae Radix Rubra* 30g, *Curcumae Radix* 15g, *Alpiniae Oxyphyllae Fructus* 12g, *Acori Tatarinowii Rhizoma* 10g, *Polygalae Radix* 6g, *Spatholobi Caulis* 12g, *Angelicae Sinensis Radix* 10g, *Salviae Miltiorrhizae Radix et Rhizoma* 15g, *Notoginseng Radix et Rhizoma* 4g, *Juglandis Semen* 20g, *Pheretima* 8g, *Hirudo* 4g, *Zingiberis Rhizoma Recens* 3 pieces, *Jujubae Fructus* 7 pieces, *Glycyrrhizae Radix et Rhizoma* 6g | Take one dose daily. Soak first, then decoct in water to yield 300 mL; divide into 2 ~ 3 doses and take. | 72.22% | Carthami Flos (9) |
| Yuan 2025 | Bushen Huayu Fang | Decoction | *Astragali Radix* 30g, *Taxilli Herba* 30g, *Eucommiae Cortex* 20g, *Polygoni Multiflori Radix* 20g, *Salviae Miltiorrhizae Radix et Rhizoma* 20g, *Angelicae Sinensis Radix* 20g, *Alpiniae Oxyphyllae Fructus* 20g, *Corni Fructus* 18g, *Dioscoreae Rhizoma* 15g, *Persicae Semen* 15g, *Acori Tatarinowii Rhizoma* 15g, *Carthami Flos* 12g, *Polygalae Radix* 12g, *Glycyrrhizae Radix et Rhizoma* 6g | Take one dose daily. Bring to a rapid boil over high heat, then simmer for 30 minutes. Prepare with 400 mL of water and take orally in two divided doses, morning and evening. | 71.43% | Persicae Semen (8) |
| Tang 2005 | Bushen Huoxue Decoction | Decoction | *Rehmanniae Radix Praeparata* 20g, *Corni Fructus* 15g, *Dioscoreae Rhizoma* 15g, *Polygoni Multiflori Radix* 20g, *Lycii Fructus* 20g, *Morindae Officinalis Radix* 20g, *Cistanches Herba* 20g, *Acori Tatarinowii Rhizoma* 15g, *Polygalae Radix* 10g, *Hirudo* 3g (Take in two divided doses), *Typhae Pollen* 15g, *Hominis Placenta* 20g, *Pseudostellariae Radix* 20g, *Citri Reticulatae Pericarpium* 10g | Take one dose daily, decoct in water, and divide it into two doses to be taken in the morning and evening. Continue for eight days, stop for two days, then resume taking it, for a total treatment period of three months. | 71.43% | Paeoniae Radix Rubra (8) |
| Wang FW 2005 | Shen Long Decoction | Decoction | *Ginseng Radix et Rhizoma* 6g, *Polygoni Multiflori Radix* 15g, *Epimedii Herba* 12g, *Pheretima* 15g, *Chuanxiong Rhizoma* 10g, *Alpiniae Oxyphyllae Fructus* 15g, *Massa Medicata Fermentata* 10g *et al.* | Take orally on an empty stomach twice daily, morning and evening. | 71.43% | Salviae Miltiorrhizae Radix et Rhizoma (7) |
| Wang FW 2004 | Shenlong Decoction | Decoction | *Ginseng Radix et Rhizoma* 6g, *Polygoni Multiflori Radix* 15g, *Epimedii Herba* 12g, *Pheretima* 15g, *Chuanxiong Rhizoma* 10g, *Alpiniae Oxyphyllae Fructus* 15g, *Massa Medicata Fermentata* 10g *et al.* | Take orally on an empty stomach twice daily, morning and evening. | 71.43% | Polygonati Rhizoma (6) |
| Wu LC 2025 | Dihuang Yinzi | Decoction | *Rehmanniae Radix Praeparata* 24g, *Corni Fructus Praeparata* 16g, *Alpiniae Oxyphyllae Fructus* 13g, *Cinnamomi Cortex* 9g, *Morindae Officinalis Radix* 17g, *Cistanches Herba* 27g, *Lycii Fructus* 19g, *Polygoni Multiflori Radix Praeparata* 11g, *Schisandrae Chinensis Fructus* 8g, *Polygalae Radix* 21g, *Acori Tatarinowii Rhizoma* 31g, *Poria* 13g, *Carthami Flos* 7g, *Lilii Bulbus* 12g, *Zingiberis Rhizoma Recens* 10g | Take one dose daily, decoct in water, and take it twice a day, once in the morning and once in the evening. | 66.67% | Lycii Fructus (6) |
| Yang HW 2025 | Jiawei Dihuang Yinzi granules | Granule | *Rehmanniae Radix Praeparata* 30g, *Polygonati Rhizoma* 15g, *Chuanxiong Rhizoma* 15g, *Polygalae Radix* 10g, *Epimedii Herba* 15g, *Dendrobii Caulis* 25g, *Poria* 15g, *Fritillariae Thunbergii Bulbus* 15g, *Acori Tatarinowii Rhizoma* 15g, *Polygoni Multiflori Radix Praeparata* 6g, *Ophiopogonis Radix* 10g, *Curcumae Radix* 20g, *Cinnamomi Cortex* 10g, *Aconiti Lateralis Radix Praeparata* 6g, *Borneolum Syntheticum* 0.3g | 12 g per sachet; take three sachets per dose, three times daily, with 300 mL of warm water. | 66.67% | Cistanches Herba (6) |
| Guo 2019 | Kidney-invigorating Blood-activating Tablets | Tablet | *Hominis Placenta* 20g, *Rehmanniae Radix Praeparata* 20g, *Chuanxiong Rhizoma* 15g, *Astragali Radix* 30g, *Acori Tatarinowii Rhizoma* 15g, *Hirudo*12g | Take orally, four tablets at a time, three times daily. | 66.67% | Epimedii Herba (6) |
| Zhao 2024 | Tongqiao Huoxue Decoction | Decoction | *Jujubae Fructus* 12g, *Zingiberis Rhizoma Recens* 9g, *Carthami Flos* 9g, *Chuanxiong Rhizoma* 15g, *Moschus* 0.15g, *Persicae Semen* 9g, *Paeoniae Radix Rubra* 12g, *Allii Fistulosi Bulbus*10g | Decoct in water and take orally, one dose per day, twice daily. | 62.50% | Dioscoreae Rhizoma (5) |
| Wu QF 2021 | Kaixin powder and Tongqiao Huoxue tang | Powder and Decoction | *Rehmanniae Radix Praeparata* 30g, *Acori Tatarinowii Rhizoma* 15g, *Polygalae Radix* 15g, *Ginkgo Folium* 15g, *Chuanxiong Rhizoma* 12g, *Ginseng Radix et Rhizoma* 10g, *Paeoniae Radix Rubra* 10g, *Arisaematis Rhizoma* 10g, *Cistanches Herba* 10g, *Corni Fructus* 10g, *Rhodiolae Crenulatae Radix et Rhizoma* 6g, *Carthami Flos* 5g, *Glycyrrhizae Radix et Rhizoma* 5g | Take one dose daily, decoct twice with conventional water, mix well, and take orally. | 61.54% | Pheretima (5) |
| Zhong 2020 | Bushen Huoxue Decoction | Decoction | *Rehmanniae Radix Praeparata* 20g, *Corni Fructus* 10g, *Alpiniae Oxyphyllae Fructus* 10g, *Chuanxiong Rhizoma* 10g, *Persicae Semen* 10g, *Carthami Flos* 10g, *Acori Tatarinowii Rhizoma* 10g, *Astragali Radix* 20g, *Atractylodis Macrocephalae Rhizoma* 15g, *Glycyrrhizae Radix et Rhizoma* 5g | Take one dose daily, decoct in water until reduced to 400 mL, and take 200 mL each morning and evening. | 60.00% | Hirudo (5) |
| Yu 2018 | Wenshen Huoxue Decoction | Decoction | *Astragali Radix* 30g, *Salviae Miltiorrhizae Radix et Rhizoma* 30g, *Puerariae Lobatae Radix* 30g, *Epimedii Herba* 10g, *Ligustri Lucidi Fructus* 10g, *Astragali Complanati Semen* 10g, *Polygalae Radix* 10g, *Chuanxiong Rhizoma* 10g, *Acori Tatarinowii Rhizoma* 10g, *Schisandrae Chinensis Fructus* 5g | Decoct in water and take one dose daily, once in the morning and once in the evening. | 60.00% | Morindae Officinalis Radix (5) |
| Hu CC 2023 | Dihuang Yinzi | Decoction | *Rehmanniae Radix Praeparata* 15g, *Cistanches Herba* 12g, *Corni Fructus* 12g, *Cinnamomi Cortex* 10g, *Morindae Officinalis Radix* 12g, *Schisandrae Chinensis Fructus* 15g, *Aconiti Lateralis Radix Praeparata* 12g, *Poria* 12g, *Ophiopogonis Radix* 15g, *Dendrobii Caulis* 12g, *Acori Tatarinowii Rhizoma* 15g, *Polygalae Radix* 12g, *Menthae Haplocalycis Herba* 9g, *Zingiberis Rhizoma Recens* 10g, *Jujubae Fructus* 5g | Decoct with water, remove dregs and take the liquid; decoct 200 mL each time, decoct twice, take warm in the morning and evening. | 60.00% | Cinnamomi Cortex (5) |
| Wang LG 2024 | Bushen Huazhuo Huoxue Decoction | Decoction | *Ginseng Radix et Rhizoma* 10g, *Astragali Radix* 20g, *Rehmanniae Radix* 10g, *Angelicae Sinensis Radix* 10g, *Rehmanniae Radix* 10g, *Poria* 10g, *Dioscoreae Rhizoma* 10g, *Salviae Miltiorrhizae Radix et Rhizoma* 10g, *Chuanxiong Rhizoma* 10g, *Glycyrrhizae Radix et Rhizoma* 3g, *Lycopi Herba* 10g, *Maydis Stigma* 30g | One dose per day, taken in two divided doses in the morning and evening, with four weeks as one course of treatment. After three courses, reduce to one dose every two days. | 54.55% | Schisandrae Chinensis Fructus (5) |
| Hu YN 2013 | FuYuan Decoction | Decoction | *Polygoni Multiflori Radix* 20g, *Acori Tatarinowii Rhizoma* 15g, *Poria* 15g, *Chuanxiong Rhizoma* 15g, *Angelicae Sinensis Radix* 12g, *Persicae Semen* 12g, *Citri Reticulatae Pericarpium* 12g, *Alpiniae Oxyphyllae Fructus* 10g, *Ginseng Radix et Rhizoma* 10g, *Corni Fructus* 10g, *Pinelliae Rhizoma* 9g, *Glycyrrhizae Radix et Rhizoma* 6g | One dose daily, decocted in water, once a day, 100 mL each time. | 50.00% | Angelicae Sinensis Radix (4) |
| Li SC 2021 | Bushen Huoxue Yizhi Decoction | Decoction | *Alpiniae Oxyphyllae Fructus* 15g, *Chuanxiong Rhizoma* 12g, *Poria* 15g, *Acori Tatarinowii Rhizoma* 12g, *Lycii Fructus* 15g, *Uncariae Ramulus cum Uncis* 15g, *Astragali Radix* 20g, *Polygonati Rhizoma* 12g, *Ophiopogonis Radix* 15g, *Polygalae Radix* 12g, *Paeoniae Radix Rubra* 15g, *Puerariae Lobatae Radix* 15g, *Persicae Semen* 9g*, Carthami Flos* 9g | Decoct in water and take 200 mL of the liquid twice daily, warm, morning and evening. | 50.00% | *Dendrobii Caulis* (4) |
| Shao 2003 | Xingnao Fusu Decoction | Decoction | *Ginseng Radix et Rhizoma* 10g, *Astragali Radix* 15g, *Rehmanniae Radix Praeparata* 20g, *Paeoniae Radix Alba* 15g, *Polygoni Multiflori Radix* 20g, *Epimedii Herba* 15g, *Salviae Miltiorrhizae Radix et Rhizoma* 15g, *Chuanxiong Rhizoma* 10g, *Gastrodiae Rhizoma* 10g, *Acori Tatarinowii Rhizoma* 10g, *Polygalae Radix* 10g | Take one dose daily, decoct in water, and take orally in two divided doses. | 45.45% | Aconiti Lateralis Radix Praeparata (4) |
| Tao 2017 | Yizhi Decoction | Decoction | *Astragali Radix* 20g, *Poria* 30g, *Atractylodis Macrocephalae Rhizoma* 15g, *Alpiniae Oxyphyllae Fructus* 12g, *Polygoni Multiflori Radix* 15g, *Bupleuri Radix* 8g, *Gastrodiae Rhizoma* 8g, *Salviae Miltiorrhizae Radix et Rhizoma* 15g, *Pheretima* 12g, *Polygalae Radix* 8g, *Acori Tatarinowii Rhizoma* 6g, *Chuanxiong Rhizoma* 15g, *Spatholobi Caulis* 15g, *Hirudo* 4g, *Citri Reticulatae Pericarpium* 8g, *Glycyrrhizae Radix et Rhizoma* 6g | Take one dose daily, decoct in water, and take orally in two divided doses. | 43.75% | *Curcumae Radix* (4) |
| Liu 2018 | Bushen Yizhi Decoction | Decoction | *Astragali Radix* 20g, *Poria* 15g, *Paeoniae Radix Rubra* 15g, *Ophiopogonis Radix* 15g, *Puerariae Lobatae Radix* 15g, *Alpiniae Oxyphyllae Fructus* 15g, *Lycii Fructus* 15g, *Uncariae Ramulus cum Uncis* 15g, *Chuanxiong Rhizoma* 12g, *Polygalae Radix* 12g, *Polygonati Rhizoma* 12g, *Acori Tatarinowii Rhizoma* 12g | One dose daily, take warm in the morning and evening. | 41.67% | Achyranthis Bidentatae Radix (2) |
| Jin 2007 | Jiannao Yizhi Capsule | Capsule | *Astragali Radix, Ligustri Lucidi Fructus, Ecliptae Herba, Polygonati Rhizoma, Achyranthis Bidentatae Radix, Lycii Fructus, Spatholobi Caulis, Cuscutae Semen, Chuanxiong Rhizoma, Epimedii Herba, Paeoniae Radix, Persicae Semen, Carthami Flos, Foeniculi Fructus, Notoginseng Radix et Rhizoma (No detailed dose indicated)* | Take 4 capsules orally, 3 times daily. | 27.27% | Rehmanniae Radix (2) |

Supplementary Table 3 Clinical efficacy evaluation criteria

| **Included study** | **Clinical efficacy evaluation criteria** |
| --- | --- |
| Dou 2023 | Efficacy criteria: Basic recovery: the patient is conscious, can perform self-care, and has essentially normal cognitive function; marked efficacy: the patient’s consciousness, self-care ability, cognitive function, and other aspects have significantly improved; ineffective: the above criteria are not met or the condition has worsened. (Basic recovery + effective) / number of cases in this group × 100.0% = overall response rate. The MMSE and ADL scales were used to evaluate patients’ cognitive function and activities of daily living. Higher scores indicate milder dementia symptoms or better ability to perform activities of daily living. |
| Guo 2019 | Markedly effective: The patient’s clinical symptoms have resolved, consciousness is clear, the patient can answer questions and solve problems independently, perform daily activities without assistance, and participate in social activities. Effective: The patient’s clinical symptoms have improved, and daily activities are basically manageable independently, but responses remain relatively slow. Ineffective: The main symptoms have not changed; the patient cannot perform daily activities independently or participate in social activities. |
| Han 2021 | Efficacy was evaluated according to the Criteria for the Diagnosis and Therapeutic Efficacy of Traditional Chinese Medicine Diseases and Syndromes. A marked response was defined as the disappearance of symptoms and signs and a ≥95% reduction in the syndrome score from baseline; a response was defined as an improvement in symptoms and signs and a ≥70% reduction in the syndrome score from baseline; and no response was defined as a ≥30% reduction in the syndrome score from baseline. Response rate = (number of markedly responsive cases + number of responsive cases) / total number of cases × 100%. |
| Hu CC 2023 | Using the MMSE scale score as the reference measure, changes in the patient’s condition were expressed as a percentage. The efficacy index was calculated as follows: (post-treatment score − pre-treatment score) / pre-treatment score × 100%. Markedly effective: efficacy index ≥ 20%; effective: efficacy index ≥ 12% and < 20%; ineffective: efficacy index < 12%. Overall response rate = (number of markedly effective cases + number of effective cases) / total number of cases × 100%. |
| Hu YN 2013 | Formulated in accordance with the Guidelines for Clinical Research on New Traditional Chinese Medicines: Markedly effective: symptom score reduced by >70% after treatment. Effective: symptom score reduced by >30%. Ineffective: symptom score reduced by less than 30%. |
| Huang 2021 | Markedly effective: MMSE score increased by ≥40%; the main symptoms basically disappeared; orientation was intact; answers were relevant; responses were prompt; the patient was independent in daily living and able to engage in general social activities. Effective: MMSE score increased by ≥20% ~<40%; some of the main symptoms disappeared; orientation was basically intact; answers were generally relevant; the patient was basically independent in daily living, but responses remained slow, with some residual impairment of intellectual function and personality. Ineffective: Cases that did not meet the above criteria. |
| Jiang Y 2014 | Markedly effective: MMSE score increased by ≥20% after treatment compared with before treatment; effective: MMSE score increased by 12%–19% after treatment compared with before treatment; ineffective: MMSE score increased by <12% after treatment compared with before treatment. Overall response rate = (markedly effective + effective) / total number of cases × 100%. |
| Jiang YY 2006 | Markedly effective: The main symptoms have essentially resolved; orientation is intact; responses are relevant; reactions are prompt; the patient is independent in daily living and able to engage in general social activities. The HDS score decreases by ≥2/3 after treatment compared with before treatment; effective: The main symptoms have partially improved; orientation is basically intact; responses are generally relevant; the patient is basically independent in daily living, but reactions remain slow, and some intellectual and personality impairments persist. The HDS score decreases by 1/3–2/3; ineffective: The main symptoms show no improvement or worsen, and the HDS score decreases by ≤1/3. |
| Jin 2007 | Following the nimodipine method, the improvement rate was calculated as (pre-treatment score − post-treatment score) / pre-treatment score × 100%. Efficacy was evaluated according to the standards in the Guidelines for Clinical Research on New Traditional Chinese Medicines and classified into five levels: clinical control, marked improvement, improvement, no effect, and deterioration. |
| Li SC 2021 | After two treatment courses, patients were evaluated using the Mini-Mental State Examination (MMSE). An increase of ≥20%, an increase of 12–19%, an increase of ≤11%, and a decrease of ≥20% in the MMSE score indicated marked efficacy, efficacy, no efficacy, and deterioration, respectively. The sum of marked efficacy and efficacy constituted the overall efficacy rate. |
| Li SG 2015 | TCM Symptom Score: In accordance with the “Guiding Principles for Clinical Research on New Chinese Medicines,” the primary symptoms are scored as 0, 3, 6, and 9 points for none, mild, moderate, and severe symptoms, respectively; secondary symptoms are scored as 0, 2, 4, and 6 points, respectively. Tongue and pulse findings are excluded. Add the scores for all items to calculate the total score and efficacy index. Efficacy index = (pre-treatment symptom score − post-treatment symptom score) / pre-treatment symptom score × 100%. |
| Li YH 2017 | Markedly effective: After treatment, the MMSE score increased by ≥40%; the patient’s major clinical symptoms essentially disappeared, orientation was intact, responses were relevant, and the patient was independent in daily living. Effective: After treatment, the MMSE score increased by ≥20%; some major clinical symptoms disappeared, orientation was basically intact, responses were generally relevant, and the patient was basically independent in daily living. Ineffective: After treatment, the MMSE score increased by <20%; major symptoms remained unchanged or worsened. Overall response rate (%) = markedly effective rate (%) + effective rate (%). |
| Liu 2018 | Efficacy evaluation: Patient efficacy was assessed after treatment. Marked improvement: MMSE score increased by ≥20%; Effective: MMSE score increased by ≥12%; Ineffective: MMSE score increased by <12%; Worsening: MMSE score decreased by ≥20%. |
| Lu 2022 | Based on the improvement in patients’ MMSE scores after treatment, the treatment efficacy rate was evaluated. Markedly effective: the post-treatment MMSE score improved by ≥5 points compared with the pretreatment score; effective: the post-treatment MMSE score improved by 1 ~ 5 points compared with the pretreatment score; ineffective: the post-treatment MMSE score improved by ≤1 point compared with the pretreatment score. The overall treatment efficacy rate = (number of markedly effective cases + number of effective cases) / total number of cases × 100%. |
| Ma 2019 | Clinical efficacy was evaluated in accordance with the Guidelines for Clinical Research of New Traditional Chinese Medicines. The efficacy index was calculated as follows: (MMSE score after treatment − MMSE score before treatment) / MMSE score before treatment × 100%. An efficacy index ≥20% was considered markedly effective; 12% ≤ efficacy index <20% was considered effective; and an efficacy index <12% was considered ineffective. The effectiveness rate was calculated as (number of markedly effective cases + number of effective cases) / number of cases in each group × 100%. |
| Man 2019 | Treatment efficacy was assessed using the Mini-Mental State Examination (MMSE-R score). Clinical control: efficacy index ≥85%; marked improvement: 50% ≤ efficacy index <85%; improvement: 20% ≤ efficacy index <50%; no change: efficacy index <20%; deterioration: post-treatment efficacy index >−20%. Efficacy index = (post-treatment score − pre-treatment score) / pre-treatment score. Response rate = (clinical control + marked improvement + improvement) / total number of cases. |
| Peng 2007 | The efficacy criteria were established with reference to the nimodipine efficacy assessment standards and the clinical efficacy assessment standards for donepezil developed by the Shanghai Institute of Mental Health. Efficacy was evaluated based on clinical symptom scores, the MMSE scale, and the BBS scale scores. Clinical control: efficacy index ≥85%; marked improvement: efficacy index ≥50%; improvement: efficacy index ≥20%; no effect: efficacy index <20%; deterioration: efficacy index ≥−20%. |
| Shao 2003 | Markedly effective: The main symptoms have essentially recovered; the patient is conscious, basically well oriented, generally responds appropriately to questions, has an ordinary response speed, and is independent in daily activities. Effective: The main psychiatric symptoms have been alleviated or have partially returned to normal; the patient generally responds appropriately to questions and is basically independent in daily activities, but responses remain slow and intellectual impairment persists. Ineffective: The main symptoms show no change.  The degree of intellectual impairment was assessed using the Mini-Mental State Examination (MMSE), and activities of daily living were assessed using the Activities of Daily Living (ADL) scale. Markedly effective: After treatment, the score increased by more than 40% or exceeded 95 points. Effective: After treatment, the score increased by 20%–40%. Ineffective: After treatment, the score increased by less than 20%.  In addition, pre- and post-treatment electroencephalographic brain maps were compared. Based on waveform changes in the brain maps, a change from moderate to normal or from severe to mild was considered markedly effective; a change from severe to moderate, moderate to mild, or mild to normal was considered effective; and no change in the brain map was considered ineffective. |
| Sheng 2009 | The overall efficacy criteria for dementia were formulated with reference to the Guiding Principles for Clinical Research on the Treatment of Dementia with Traditional Chinese Medicines (New Drugs). The MMSE score was used as the primary reference indicator, and the nimodipine method was applied for calculation. Formula: [(Score after treatment − Score before treatment) ÷ Score before treatment] × 100%, expressed as a percentage.  Markedly effective: Symptoms and signs improved significantly, and the syndrome score decreased by ≥70%.  Improved: Symptoms and signs both improved, and the syndrome score decreased by ≥30%.  Ineffective: Symptoms and signs showed no significant improvement or even worsened, and the syndrome score decreased by less than 30%. |
| Tang 2005 | Based on the Criteria for the Diagnosis, Syndrome Differentiation, Classification, and Efficacy Assessment of Senile Dementia. Markedly effective: The main symptoms have essentially disappeared; the patient is lucid, fully oriented, answers questions correctly, responds promptly, and is able to manage daily activities independently. Effective: The main psychiatric symptoms have weakened or partially disappeared; the patient is basically able to manage daily activities independently and generally answers questions correctly, but responses are slow, and some intellectual and personality impairments remain. Ineffective: The main symptoms show no improvement or have worsened; the patient is unable to manage daily activities independently and is demented. |
| Tao 2017 | With reference to the Guiding Principles for Clinical Research of New Traditional Chinese Medicines, the severity of the main symptoms—such as soreness and weakness of the lower back and knees, dizziness, tinnitus and hearing loss, and limb numbness—was assessed on a scale of 0–3: 0, no symptoms; 1, mild and tolerable symptoms; 2, severe symptoms affecting daily life; 3, extremely severe symptoms. Higher scores indicate more severe symptoms. Clinical recovery: symptoms disappeared and the syndrome score decreased by ≥95%; marked improvement: symptoms improved significantly and the syndrome score decreased by ≥70%; improvement: symptoms improved and the syndrome score decreased by ≥30%; ineffective: the syndrome score decreased by <30%, with no significant improvement or even worsening of symptoms and signs. |
| Wang FW 2004 | Using the nimodipine method, namely: (pre-treatment score − post-treatment score) / pre-treatment score × 100%. Efficacy evaluation criteria: According to the newly revised standards in the “Guiding Principles for Clinical Research on the Treatment of Dementia with New Drugs (Traditional Chinese Medicine)” from the “Guiding Principles for Clinical Research on New Traditional Chinese Medicines” issued by the Ministry of Health, efficacy is classified into five levels: clinical basic control, marked improvement, improvement, no effect, and deterioration. |
| Wang FW 2005 | Using the nimodipine method, namely: (pre-treatment score − post-treatment score) / pre-treatment score × 100%. Efficacy evaluation criteria: According to the newly revised standards in the “Guiding Principles for Clinical Research on the Treatment of Dementia with New Drugs (Traditional Chinese Medicine)” from the “Guiding Principles for Clinical Research on New Traditional Chinese Medicines” issued by the Ministry of Health, efficacy is classified into five levels: clinical basic control, marked improvement, improvement, no effect, and deterioration. |
| Wang LG 2024 | Markedly effective: The patient’s main clinical symptoms have essentially disappeared, and cognitive function, mental status, and activities of daily living have returned to normal. Effective: The patient’s main clinical symptoms have lessened or partially disappeared, but cognitive function has not fully recovered; the patient remains slow to respond but can perform some activities of daily living. Ineffective: The patient’s main clinical symptoms show no improvement or have even worsened; the patient is mentally confused and in poor condition, with no basic ability to care for themselves. |
| Wang SF 2015 | For each patient, MMSE scores were assessed before and after treatment. Treatment efficacy was calculated as the difference between the post-treatment and pre-treatment scores: a score difference >5 indicated significant improvement, 2 ≤ score difference <5 indicated improvement, −1 ≤ score difference <2 indicated no effect, and a score difference <−1 indicated deterioration. |
| Wang TS 2016 | Clinical control: The patient’s main symptoms have essentially returned to normal, orientation is intact, questions are answered correctly, the patient is capable of self-care, and can resume ordinary social activities; marked improvement: most of the patient’s main symptoms have returned to normal, orientation is basically intact, questions are answered essentially correctly, responses are generally adequate, and the patient is capable of self-care; improvement: the patient’s main psychiatric symptoms have been alleviated or partially resolved, the patient is basically capable of self-care and answers questions essentially correctly, but responses remain slow, with persistent intellectual and personality impairments; no improvement: the patient’s main symptoms remain unchanged or continue to worsen. The overall response rate was calculated as clinical control + marked improvement + improvement. |
| Wu LC 2025 | Markedly effective: The patient’s symptoms completely disappear after treatment, they are fully independent in daily living, and can participate normally in social activities; effective: the patient’s symptoms improve significantly after treatment, they are basically independent in daily living, and their social activities are not affected; ineffective: after treatment, the patient still experiences symptoms such as memory decline and low mood, is unable to live independently, and cannot engage in normal social activities. |
| Wu QF 2021 | Markedly effective: efficacy index ≥20%; effective: efficacy index 12%–19%; ineffective: efficacy index −12%–11%; worsened: efficacy index <−12%. Efficacy index = (post-treatment MMSE score − pre-treatment MMSE score) / pre-treatment MMSE score × 100%. |
| Yang HW 2025 | The efficacy criteria for TCM syndromes were established in accordance with the Guiding Principles for Clinical Research of New Traditional Chinese Medicines (Trial Implementation). Efficacy index (%) = (pre-treatment score − post-treatment score) ÷ pre-treatment score × 100%. Markedly effective: efficacy index ≥70%; effective: efficacy index <70% and ≥30%; ineffective: efficacy index <30%. Overall response rate (%) = (number of markedly effective cases + number of effective cases) ÷ total number of cases × 100%. |
| Yang P 2019 | Patients’ cognitive function was assessed before and after treatment using the revised Folstein Mini-Mental State Examination (MMSE). Clinical efficacy was evaluated using three grades: markedly effective, improved, and ineffective: (1) markedly effective: the post-treatment MMSE score increased by ≥40% compared with the pretreatment score; (2) improved: an increase of ≥20%; (3) ineffective: an increase of <20%. The response rate was calculated as the markedly effective rate plus the improvement rate. |
| Yao H 2017 | Developed according to the criteria in the Introduction to the Diagnostic Criteria and Assessment Tools for Vascular Dementia. Evaluation was based on changes in the patient’s syndrome-related symptom scores: 1 indicates marked improvement, 2 moderate improvement, 3 slight improvement, 4 no change, 5 slight deterioration, 6 moderate deterioration, and 7 marked deterioration. Basic control: efficacy index ≥85%; significant improvement: 66% ≤ efficacy index <85%; effective: 33% ≤ efficacy index <66%; ineffective: efficacy index <33%. Efficacy index = (pre-treatment score − post-treatment score) / pre-treatment score × 100%. Response rate = (basic control + significant improvement + effective) / n × 100%. |
| Yao YJ 2021 | The efficacy of the two groups was assessed using the HDS and MMSE. An increase of >10 points in the HDS score or ≥40% in the MMSE score was considered markedly effective; an increase of 6–10 points in the HDS score or 20%–39% in the MMSE score was considered effective; results not meeting the above criteria were considered ineffective. The effective and markedly effective cases were included in the overall efficacy rate. |
| Yu 2018 | Before treatment and three months after treatment, the National Institutes of Health Stroke Scale (NIHSS) was used to assess patients’ neurological deficits; a higher score indicated more severe neurological impairment. The Activities of Daily Living (ADL) scale was used to evaluate patients’ ability to perform daily activities; a lower score indicated poorer functional capacity. According to the Criteria for the Diagnosis, Syndrome Differentiation, and Efficacy Evaluation of Vascular Dementia, combined with the Mini-Mental State Examination (MMSE), clinical efficacy was assessed. The efficacy index was calculated as the difference between the post-treatment and pretreatment scores divided by the pretreatment score, expressed as a percentage. Markedly effective: efficacy index ≥20%; effective: efficacy index ≥12%; ineffective: efficacy index <12%. The overall response rate = (markedly effective + effective) / total number of cases × 100%. |
| Yuan 2025 | The grading criteria were based on the methods specified in the Guidelines for Clinical Research on New Traditional Chinese Medicines. Clinical cure: all clinical manifestations related to kidney essence deficiency disappeared completely, and the TCM syndrome score decreased by ≥95%; marked efficacy: clinical symptoms were significantly alleviated, and the syndrome score decreased by ≥70%; improvement: symptoms were somewhat relieved, and the score decreased by ≥30%; ineffective: none of the above criteria were met. The overall response rate = clinical cure rate + marked efficacy rate + improvement rate. |
| Zhao 2024 | Based on the Mini-Mental State Examination (MMSE), the results were assessed as follows: markedly effective: clinical symptoms were significantly relieved, with an increase in MMSE scores of >20%; effective: clinical symptoms improved, with an increase in MMSE scores of 12%–20%; ineffective: the degree of improvement did not meet the above criteria. |
| Zheng 2009 | According to the efficacy evaluation criteria of the 2002 "Criteria for Diagnosis, Syndrome Differentiation, and Efficacy Evaluation of Vascular Dementia (for research use)" issued by the Internal Medicine Committee on Anti-Aging of the China Association of Chinese Medicine. |
| Zhong 2020 | Efficacy was evaluated using the reduction rate in the MMSE score: Efficacy index = (pre-treatment score − post-treatment score) / pre-treatment score × 100%. Cured: efficacy index ≥75%; markedly effective: 75% > efficacy index ≥50%; effective: 50% > efficacy index ≥25%; ineffective: efficacy index <25%. |
| Zhu 2015 | Dementia improvement was assessed using the Hasegawa Dementia Scale (HDS). Compared with the pretreatment score, an HDS score >31 after treatment or an increase of 10 points was considered markedly effective; a post-treatment score >22 and ≤31 or an increase of ≥6 and <10 points was considered effective; a post-treatment score >10 and ≤22, an increase of less than 6 points, or worsening was considered ineffective. The total effective rate = markedly effective + effective. |

## 2.3 PRISMA 2020 checklist

| **Section and Topic** | **Item #** | **Checklist item** | **Location where item is reported** |
| --- | --- | --- | --- |
| **TITLE** | | |  |
| Title | 1 | Identify the report as a systematic review. | Title page |
| **ABSTRACT** | | |  |
| Abstract | 2 | See the PRISMA 2020 for Abstracts checklist. | Abstract |
| **INTRODUCTION** | | |  |
| Rationale | 3 | Describe the rationale for the review in the context of existing knowledge. | 1 Introduction |
| Objectives | 4 | Provide an explicit statement of the objective(s) or question(s) the review addresses. | 1 Introduction |
| **METHODS** | | |  |
| Eligibility criteria | 5 | Specify the inclusion and exclusion criteria for the review and how studies were grouped for the syntheses. | 2.3 Eligibility criteria  2.4 Exclusion criteria |
| Information sources | 6 | Specify all databases, registers, websites, organisations, reference lists and other sources searched or consulted to identify studies. Specify the date when each source was last searched or consulted. | 2.2 Search strategy |
| Search strategy | 7 | Present the full search strategies for all databases, registers and websites, including any filters and limits used. | 2.2 Search strategy |
| Selection process | 8 | Specify the methods used to decide whether a study met the inclusion criteria of the review, including how many reviewers screened each record and each report retrieved, whether they worked independently, and if applicable, details of automation tools used in the process. | 2.5 Selection process |
| Data collection process | 9 | Specify the methods used to collect data from reports, including how many reviewers collected data from each report, whether they worked independently, any processes for obtaining or confirming data from study investigators, and if applicable, details of automation tools used in the process. | 2.6 Data collection process |
| Data items | 10a | List and define all outcomes for which data were sought. Specify whether all results that were compatible with each outcome domain in each study were sought (e.g. for all measures, time points, analyses), and if not, the methods used to decide which results to collect. | 2.8 Data synthesis and statistical analysis |
|  | 10b | List and define all other variables for which data were sought (e.g. participant and intervention characteristics, funding sources). Describe any assumptions made about any missing or unclear information. | 2.8 Data synthesis and statistical analysis |
| Study risk of bias assessment | 11 | Specify the methods used to assess risk of bias in the included studies, including details of the tool(s) used, how many reviewers assessed each study and whether they worked independently, and if applicable, details of automation tools used in the process. | 2.7 Study risk of bias assessment |
| Effect measures | 12 | Specify for each outcome the effect measure(s) (e.g. risk ratio, mean difference) used in the synthesis or presentation of results. | 2.8 Data synthesis and statistical analysis |
| Synthesis methods | 13a | Describe the processes used to decide which studies were eligible for each synthesis (e.g. tabulating the study intervention characteristics and comparing against the planned groups for each synthesis (item #5)). | 2.8 2.9 2.10 |
|  | 13b | Describe any methods required to prepare the data for presentation or synthesis, such as handling of missing summary statistics, or data conversions. | 2.8 2.9 2.10 |
|  | 13c | Describe any methods used to tabulate or visually display results of individual studies and syntheses. | 2.8 2.9 2.10 |
|  | 13d | Describe any methods used to synthesize results and provide a rationale for the choice(s). If meta-analysis was performed, describe the model(s), method(s) to identify the presence and extent of statistical heterogeneity, and software package(s) used. | 2.8 2.9 2.10 |
|  | 13e | Describe any methods used to explore possible causes of heterogeneity among study results (e.g. subgroup analysis, meta-regression). | 2.8 2.9 2.10 |
|  | 13f | Describe any sensitivity analyses conducted to assess robustness of the synthesized results. | 2.8 2.9 2.10 |
| Reporting bias assessment | 14 | Describe any methods used to assess risk of bias due to missing results in a synthesis (arising from reporting biases). | 2.11 Assessment of Reporting Biases |
| Certainty assessment | 15 | Describe any methods used to assess certainty (or confidence) in the body of evidence for an outcome. | 2.8 Data synthesis and statistical analysis |
| **RESULTS** | | |  |
| Study selection | 16a | Describe the results of the search and selection process, from the number of records identified in the search to the number of studies included in the review, ideally using a flow diagram. | 3.1 Study selection |
|  | 16b | Cite studies that might appear to meet the inclusion criteria, but which were excluded, and explain why they were excluded. | 3.1 Study selection |
| Study characteristics | 17 | Cite each included study and present its characteristics. | 3.2 Study characteristics |
| Risk of bias in studies | 18 | Present assessments of risk of bias for each included study. | 3.3 Risk of bias in studies |
| Results of individual studies | 19 | For all outcomes, present, for each study: (a) summary statistics for each group (where appropriate) and (b) an effect estimate and its precision (e.g. confidence/credible interval), ideally using structured tables or plots. | 3.4 3.5 |
| Results of syntheses | 20a | For each synthesis, briefly summarise the characteristics and risk of bias among contributing studies. | 3.4 3.5 |
|  | 20b | Present results of all statistical syntheses conducted. If meta-analysis was done, present for each the summary estimate and its precision (e.g. confidence/credible interval) and measures of statistical heterogeneity. If comparing groups, describe the direction of the effect. | 3.4 3.5 |
|  | 20c | Present results of all investigations of possible causes of heterogeneity among study results. | 3.4 3.5 |
|  | 20d | Present results of all sensitivity analyses conducted to assess the robustness of the synthesized results. | 3.4 3.5 |
| Reporting biases | 21 | Present assessments of risk of bias due to missing results (arising from reporting biases) for each synthesis assessed. | 3.6 |
| Certainty of evidence | 22 | Present assessments of certainty (or confidence) in the body of evidence for each outcome assessed. | 3.6 |
| **DISCUSSION** | | |  |
| Discussion | 23a | Provide a general interpretation of the results in the context of other evidence. | 4 Discussion |
|  | 23b | Discuss any limitations of the evidence included in the review. | 5 Limitations |
|  | 23c | Discuss any limitations of the review processes used. | 5 Limitations |
|  | 23d | Discuss implications of the results for practice, policy, and future research. | 4 5 |
| **OTHER INFORMATION** | | |  |
| Registration and protocol | 24a | Provide registration information for the review, including register name and registration number, or state that the review was not registered. | 2.1 Protocol registration |
|  | 24b | Indicate where the review protocol can be accessed, or state that a protocol was not prepared. | 12 Data Availability Statement |
|  | 24c | Describe and explain any amendments to information provided at registration or in the protocol. | 2.1 Protocol registration |
| Support | 25 | Describe sources of financial or non-financial support for the review, and the role of the funders or sponsors in the review. | 9 Funding |
| Competing interests | 26 | Declare any competing interests of review authors. | 7 Conflict of Interest |
| Availability of data, code and other materials | 27 | Report which of the following are publicly available and where they can be found: template data collection forms; data extracted from included studies; data used for all analyses; analytic code; any other materials used in the review. | 12 Data Availability Statement |

*From:*  Page MJ, McKenzie JE, Bossuyt PM, Boutron I, Hoffmann TC, Mulrow CD, et al. The PRISMA 2020 statement: an updated guideline for reporting systematic reviews. BMJ 2021;372:n71. doi: 10.1136/bmj.n71
